# Optimal entanglement distribution policies in homogeneous repeater chains with cutoffs

**DOI:** 10.1038/s41534-023-00713-9

**Published:** 2023-05-06

**Authors:** Álvaro G. Iñesta, Gayane Vardoyan, Lara Scavuzzo, Stephanie Wehner

**Affiliations:** 1 QuTech, Delft University of Technology, Delft, The Netherlands; 2 EEMCS, Delft University of Technology, Delft, The Netherlands; 3 Kavli Institute of Nanoscience, Delft University of Technology, Delft, The Netherlands

**Keywords:** Quantum information, Computer science, Information technology

## Abstract

We study the limits of bipartite entanglement distribution using a chain of quantum repeaters that have quantum memories. To generate end-to-end entanglement, each node can attempt the generation of an entangled link with a neighbor, or perform an entanglement swapping measurement. A maximum storage time, known as cutoff, is enforced on the memories to ensure high-quality entanglement. Nodes follow a policy that determines when to perform each operation. Global-knowledge policies take into account all the information about the entanglement already produced. Here, we find global-knowledge policies that minimize the expected time to produce end-to-end entanglement. Our methods are based on Markov decision processes and value and policy iteration. We compare optimal policies to a policy in which nodes only use local information. We find that the advantage in expected delivery time provided by an optimal global-knowledge policy increases with increasing number of nodes and decreasing probability of successful swapping.

## Introduction

Bipartite entangled states shared between two parties are often required as a basic resource in quantum network applications. As an example, in cryptography, bipartite entanglement can be directly used for quantum key distribution between two parties^1,2^, but also in multi-party applications such as quantum secret sharing^3^. Bipartite entanglement can also be used to generate multipartite entangled states that are necessary for other applications^4–6^. As a consequence, a reliable method to distribute entanglement in a quantum network is crucial for the implementation of quantum cryptography applications.

**Fig. 1:**
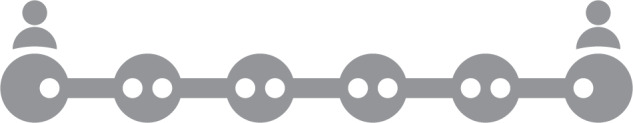
A quantum repeater chain that can store two qubits per intermediate node and one qubit per end node. White circles represent qubits. All nodes are equidistant and identical.

Two neighboring nodes in a quantum network can generate a shared bipartite entangled state, which we call an entangled link. This can be done, e.g., by generating an entangled pair at one node and sending half of the pair to the neighbor via an optical fiber^7,8^ or free space^9,10^. Two distant nodes can generate an entangled link by generating entanglement between each pair of adjacent nodes along a path that connects them, and then combining these entangled links into longer-distance bipartite entanglement via entanglement swap operations^11,12^. This path constitutes a quantum repeater chain (see Fig. 1). We consider repeater chains in which nodes can store quantum states in the form of qubits and perform operations and measurements on them. Experimentally, qubits can be realized with different technologies, such as NV centers^13–17^ and trapped ions^18,19^.

We focus on a single repeater chain of *n* equidistant and identical nodes, which could be part of a larger quantum network. To generate an entangled link between the two end nodes, also called end-to-end entanglement, we assume the nodes can perform the following operations: (i) heralded generation of entanglement between neighbors^13,20^, which succeeds with probability *p* and otherwise raises a failure flag; (ii) entanglement swaps^11,12,21^, which consume two adjacent entangled links to generate a longer-distance link with probability *p*_s_; and (iii) removal of any entangled link that existed for longer than some cutoff time *t*_cut_, to prevent generation of low-quality end-to-end entanglement due to decoherence^16,22–25^. Note that cutoff times are a key ingredient, since many applications require quantum states with a high enough quality.

We assume that nodes always attempt entanglement generation if there are qubits available. Cutoffs are always applied whenever an entangled link becomes too old. However, nodes are free to attempt swaps as soon as entangled links are available or some time later, so they must agree on an entanglement distribution policy: a set of rules that indicate when to perform a swap. We define an optimal policy as a policy that minimizes the expected entanglement delivery time, which is the average time required to generate end-to-end entanglement. Here, we consider optimal global-knowledge policies, in which nodes have information about all the entangled links in the chain. A policy is local when the nodes only need to know the state of the qubits they hold. An example of local policy is the swap-asap policy, in which each node performs a swap as soon as both entangled links are available.

Previous work on quantum repeater chains has mostly focused on the analysis of specific policies rather than on the search for optimal policies. For example, ref. ^26^ provides analytical bounds on the delivery time of a “nested” policy^27^, and ref. ^28^ optimizes the parameters of such a policy with a dynamic programming approach. Delivery times can be studied using Markov models. In ref. ^29^, the authors introduce a methodology based on Markov chains to calculate the expected delivery time in repeater chains that follow a particular policy. Similar techniques have also been applied to other quantum network topologies, such as the quantum switch^30,31^. Here, we focus on Markov decision processes (MDPs), which have already been applied to related problems, e.g., in ref. ^32^, the authors use an MDP formulation to maximize the quality of the entanglement generated between two neighboring nodes and between the end nodes in a three-node repeater chain. Our work builds on ref. ^33^, wherein the authors find optimal policies for quantum repeater chains with perfect memories. Since quantum memories are expected to be noisy, particularly in the near future, quantum network protocols must be suitable for imperfect memories. Here, we take a crucial step towards the design of high-quality entanglement distribution policies for noisy hardware. By formulating a generalized MDP to include finite storage times, we are able to find optimal policies in quantum repeater chains with imperfect memories. Our optimal policies provide insights for the design of entanglement distribution protocols.

Our main contributions are as follows:

- We introduce a general MDP model for homogeneous repeater chains with memory cutoffs. The latter constraint poses a previously unaddressed challenge: MDP states must incorporate not only entangled link absence/presence, but also link age;
- We find optimal policies for minimizing the expected end-to-end entanglement delivery time, by solving the MDP via value and policy iteration;
- Our optimal policies take into account global knowledge of the state of the chain and therefore constitute a lower bound to the expected delivery time of policies that use only local information.

Our main findings are as follows:

- The optimal expected delivery time in a repeater chain with deterministic swaps (*p*_s_ = 1) can be orders of magnitude smaller than with probabilistic swaps;
- When swaps are deterministic, the advantage in expected delivery time offered by an optimal policy as compared to the swap-asap policy increases for lower probability of entanglement generation, *p*, and lower cutoff time, *t*_cut_, in the parameter region explored. However, when swaps are probabilistic, we find the opposite behavior: the advantage increases for higher *p* and *t*_cut_;
- The advantage provided by optimal policies increases with higher number of nodes, both when swaps are deterministic and probabilistic, albeit the advantage is larger in case of the latter.

## Results

### Network model

We analyze quantum repeater chains wherein nodes can store quantum states in the form of qubits and can perform three basic operations with them: entanglement generation, entanglement swaps, and cutoffs.

Two adjacent nodes can attempt the heralded generation of an entangled link (i.e., a shared bipartite entangled state), succeeding with probability *p*. Generation of entanglement is heralded, meaning that the nodes receive a message stating whether they successfully generated an entangled link or not^13,20^. We assume that entanglement generation is noisy. Hence, the newly generated entangled links are not maximally entangled states but Werner states^34^. Werner states are maximally entangled states that have been subjected to a depolarizing process, which is a worst-case noise model^35^, and they can be written as follows:

1 $$\rho =\frac{4F-1}{3}\left\vert {\phi }^{+}\right\rangle \left\langle {\phi }^{+}\right\vert +\frac{1-F}{3}{{\mathbb{I}}}_{4},$$

where $\left\vert {\phi }^{+}\right\rangle =\frac{\left\vert 00\right\rangle +\left\vert 11\right\rangle }{\sqrt{2}}$ is a maximally entangled state, *F* is the fidelity of the Werner state to the state $\left\vert {\phi }^{+}\right\rangle$, and ${{\mathbb{I}}}_{d}$ is the *d*-dimensional identity. In our notation, the fidelity of a mixed state *ρ* to a pure state $\left\vert \phi \right\rangle$ is defined as

2 $$F(\rho ,\left\vert \phi \right\rangle ):= \left\langle \phi \right\vert \rho \left\vert \phi \right\rangle .$$

We assume that the fidelity of newly generated entangled links is *F*_new_ ≤ 1.

**Fig. 2:**
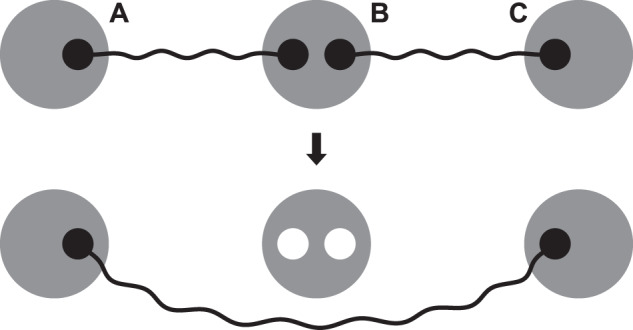
Entanglement swap. When node B performs a swap, an entangled link between nodes A and B and an entangled link between nodes B and C are consumed to produce a single entangled link between A and C. This operation is essential for the generation of long-distance entanglement.

Two neighboring entangled links can be fused into a longer-distance entangled link via entanglement swapping. Consider a situation where node B shares an entangled link with node A, and another link with node C (see Fig. 2). Then, B can perform an entanglement swap to produce an entangled link between A and C while consuming both initial links^11,12,21^. We refer to the link generated in a swap operation as a swapped link. This operation is also probabilistic: a new link is produced with probability *p*_s_, and no link is produced (but both input links are still consumed) with probability 1 − *p*_s_.

The generation of an entangled link between two end nodes without intermediate repeaters is limited by the distance between the end nodes^36^—e.g., the noise affecting a photon sent over an optical fiber grows exponentially with the length of the fiber^27^. Therefore, a repeater chain that makes use of entanglement swapping is needed to generate end-to-end entanglement over long distances.

The fidelity of a quantum state decreases over time due to couplings to the environment^35,37^. These decoherence processes can be captured using a white noise model in which a depolarizing channel is applied to the entangled state at every instant. As a result, the fidelity of a Werner state at time *t*, *F*(*t*), is given by

3 $$F(t)=\frac{1}{4}+\left(F(t-\Delta t)-\frac{1}{4}\right){{{{\rm{e}}}}}^{-\Delta t/\tau },$$

where Δ*t* is an arbitrary interval of time and *τ* is a parameter that characterizes the exponential decay in fidelity of the whole entangled state due to the qubits being stored in noisy memories. This parameter depends on the physical realization of the qubit. (3) is derived in Supplementary Note 1.

In general, quantum network applications require quantum states with fidelity above some threshold value ${F}_{\min }$. A common solution is to impose a cutoff time *t*_cut_ on the entangled links: all entangled links used to generate the final end-to-end link must be generated within a time window of size *t*_cut_^23^. Imposing memory cutoffs requires keeping track of the time passed since the creation of each entangled link. We call this time the age of the link. A link is discarded whenever it gets older than *t*_cut_. Moreover, we assume that an entangled link generated as a result of entanglement swapping assumes the age of the oldest link that was involved in the swapping operation. Another valid approach to calculate the age of a swapped link would be to re-compute the age based on the post-swap fidelity, although this would lead to a more complicated formulation to ensure that all the links that were used to produce a swapped link were generated within the time window of size *t*_cut_. To produce end-to-end links with fidelity above ${F}_{\min }$ on a repeater chain that generates new links with fidelity *F*_new_, it suffices to ensure that the sequence of events that produces the lowest end-to-end fidelity satisfies this requirement. In Supplementary Note 2, we show that such a sequence of events corresponds to all links being simultaneously generated in the first attempt and all the entanglement swaps being performed at the end of the *t*_cut_ interval. Analyzing such a sequence of events leads to the following condition for the cutoff time:

4 $${t}_{{{{\rm{cut}}}}}\le -\tau \ln \left(\frac{3}{4{F}_{{{{\rm{new}}}}}-1}{\left(\frac{4{F}_{\min }-1}{3}\right)}^{\frac{1}{n-1}}\right),$$

where *n* is the number of nodes. For a full derivation of the previous condition, see Supplementary Note 2.

In this paper, we consider quantum networks that operate with a limited number of qubits. Specifically, we use the following additional assumptions:

(i) The chain is homogeneous, i.e., the hardware is identical in all nodes. This means that all pairs of neighbors generate links with the same success probability *p* and fidelity *F*_new_, all swaps succeed with probability *p*_s_, all states decohere according to some coherence time *τ*, and all nodes apply the same cutoff time *t*_cut_. This assumption may not hold for some long-distance quantum networks where each node is implemented using a different technology, but may be directly applicable to, e.g., small metropolitan-scale networks.
(ii) We assume that each node has only two storage qubits, each of which is used to generate entanglement with one side of the chain. Each end node has a single storage qubit. This assumption is in line with the expectations for early quantum networks, in which nodes are likely to have a number of storage qubits on the order of the unit (e.g., in ref. ^17^ the authors realized the first three-node quantum network using NV centers, each with a single storage qubit).
(iii) We also assume that classical communication between nodes is instantaneous. This means that every node has global knowledge of the state of the repeater chain in real time. In general, this is not a realistic assumption. However, given that classical communication delays decrease the performance of the network, our results constitute a lower bound on the expected delivery time of real setups and can be used as a benchmark.
(iv) Time is discretized into non-overlapping time slots. During one time slot: (*i*) first, each pair of neighboring nodes attempts entanglement generation if they have free qubits; (*ii*) second, some time is allocated for the nodes to attempt entanglement swaps; and (*iii*) lastly nodes discard any entangled link that existed for longer than *t*_cut_ time slots. To decide if they want to perform a swap in the second part of the time step, nodes can take into account the state of the whole chain, including the results from entanglement generation within the same time slot, since classical communication is instantaneous. The unit of time used in this paper is the duration of a time slot, unless otherwise specified.

A repeater chain under the previous assumptions is characterized by four parameters:

- ⋅ *n*: number of nodes in the chain, including end nodes.
- ⋅ *p*: probability of successful entanglement generation.
- ⋅ *p*_s_: probability of successful swap.
- ⋅ *t*_cut_: cutoff time. Note that *F*_new_, ${F}_{\min }$, and *τ* are used to determine a proper value of cutoff time (see condition (4)), but they are not needed after that.

In an experimental setup, the value of *p* is determined by the inter-node distance and the type of hardware used, as quantum nodes can be realized using different technologies, such as NV centers^13–17^ and trapped ions^18,19^. Linear optics setups generally perform swaps with probability *p*_s_ = 0.5^11,38^, while other setups can perform deterministic swaps (*p*_s_ = 1) at the cost of a slower speed of operation^17^. The cutoff time *t*_cut_ can be chosen by the user, as long as condition (4) is satisfied. Note that (4) depends on *τ* (which depends on the hardware available), *F*_new_ (which depends on the hardware and the choice of entanglement generation protocol), and ${F}_{\min }$ (which is specified by the final application).

**Fig. 3:**
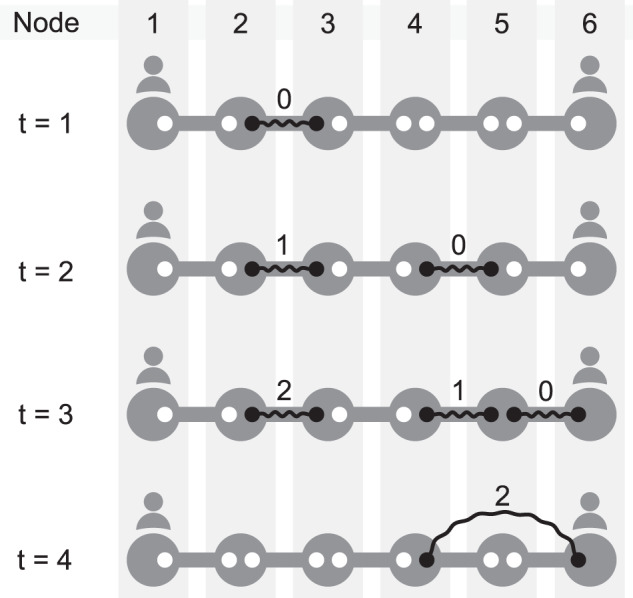
Example of entangled link dynamics in a repeater chain. Each row represents the state of the chain at the end of time slot *t*. Entangled links are represented as black solid lines, with occupied qubits as black circles. The number above each entangled link is the age of the link. We assume cutoff *t*_cut_ = 3.

The state of the repeater chain at the end of each time slot can be described using the age of every entangled link. In Fig. 3 we show an example of the evolution of the state of a chain with cutoff *t*_cut_ = 3, over four time slots:

- In the first time slot (*t* ∈ [0, 1)), all pairs of neighbors attempt entanglement generation, but it only succeeds between nodes two and three. No swaps can be performed, and the only link present is younger than the cutoff, so it is not discarded.
- In the second time slot (*t* ∈ [1, 2)), the age of the link between nodes two and three increases by one. All pairs of neighbors (except nodes two and three) attempt entanglement generation, which succeeds between nodes four and five.
- In the third time slot (*t* ∈ [2, 3)), the age of both existing links increases by one. All pairs of neighbors (except nodes two and three and nodes four and five) attempt entanglement generation, and only nodes five and six succeed. A swap can be performed at node five but they decide to wait.
- In the fourth time slot (*t* ∈ [3, 4)), the age of every existing link increases by one. Nodes one and two and nodes three and four attempt entanglement generation but none of the pairs succeeds. A swap is successfully performed at node five, and a new link between nodes four and six is generated. This new link assumes the age of the oldest link involved in the swap operation. Lastly, the entangled link between nodes two and three is discarded, as its age reached the cutoff time.

### Optimal entanglement distribution policies

As described above, nodes always attempt entanglement generation if there are qubits available. Cutoffs are always applied whenever an entangled state becomes too old. Since nodes are free to attempt swaps as soon as entangled links are available or sometime later, they must agree on an entanglement distribution policy: a set of rules that indicate when to perform a swap. An optimal policy minimizes the average time required to generate end-to-end entanglement when starting from any state (i.e., from any combination of existing links) and following said policy. In particular, it minimizes the mean entanglement delivery time, which is the average time required to generate end-to-end entanglement when starting from the state with no entangled links. We employ the mean entanglement delivery time as a performance metric.

In a global-knowledge policy, nodes have information about all the entangled links in the chain. In a local-knowledge policy, the nodes only need to know the state of the qubits they hold. An example of local policy is the swap-asap policy, in which each node performs a swap as soon as both entangled links are available.

We model the evolution of the state of the repeater chain as an MDP. We then formulate the Bellman equations^39^ and solve them using value iteration and policy iteration to find global-knowledge optimal policies. More details and formal definitions are provided in the Methods Section.

**Fig. 4:**
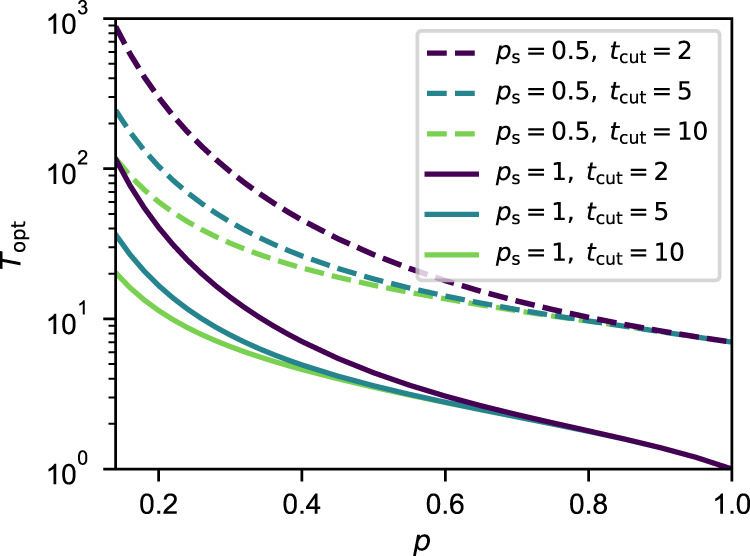
The expected delivery time increases with lower *p*, *p*_s_, and *t*_cut_. Expected delivery time of an optimal policy, *T*_opt_, versus *p* in a five-node chain, for different values of cutoff (*t*_cut_ = 2, 5, 10). Solid lines correspond to deterministic swaps (*p*_s_ = 1) and dashed lines correspond to probabilistic swaps with *p*_s_ = 0.5.

Let us now describe the relation between the expected delivery time of an optimal policy, *T*_opt_, and the variables of the system (*n*, *p*, *p*_s_, and *t*_cut_). Repeater chains with a larger number of nodes *n* yield a larger *T*_opt_, since more entangled links need to be generated probabilistically. When *p* is small, more entanglement generation attempts are required to succeed, yielding a larger *T*_opt_. Decreasing *p*_s_ also increases *T*_opt_, since more attempts at entanglement swapping are required on average. When *t*_cut_ is small, all entangled states must be generated within a small time window and therefore *T*_opt_ is also larger. Figure 4 shows the expected delivery time of an optimal policy in a five-node chain. Interestingly, *p*_s_ has a much stronger influence on *T*_opt_ than *p* and *t*_cut_: decreasing *p*_s_ from 1 to 0.5 in a five-node chain translates into an increase in *T*_opt_ of an order of magnitude. Similar behavior is observed for other values of *n*, as shown in Supplementary Note 3.

**Fig. 5:**
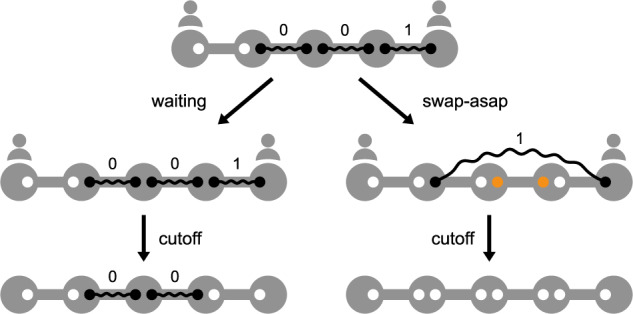
Swap-asap policies free up qubits, but swapped links expire earlier. Evolution of an example state when following a waiting policy versus the swap-asap policy during a single time slot. Entangled links are represented as solid black lines, with occupied qubits in black and free qubits in white. A waiting policy decides to not perform any swap, while the swap-asap policy decides to swap all three links. The swap frees up qubits (marked in orange) that can be used to resume entanglement generation either if the swap is successful, as in the picture, or not. After performing swaps, a cutoff *t*_cut_ = 1 is applied and links with age 1 are removed, causing the swapped link to expire.

To evaluate the advantages of an optimal policy, we use the swap-asap policy as a baseline. Early swaps can provide an advantage in terms of delivery time, since swapping earlier can free up qubits that can be used to generate backup entangled links, as displayed in the first transition in Fig. 5. However, the age of a swapped link may reach the cutoff time earlier than one of the input links consumed in the swap, as the swapped link assumes the age of the oldest input link. Following the example in Fig. 5 and assuming *t*_cut_ = 1, if no swaps are performed, the links between nodes two and three and between three and four will exist for one more time slot, while the link between nodes four and five will be removed immediately since it reached the cutoff time. If both swaps are performed, the swapped link between nodes two and five will be removed immediately since it reached the cutoff time. Since we have arguments in favor of and against swapping early, it is not trivial to determine the scenarios in which the swap-asap policy is close to optimal. Next, we compare the expected delivery times of an optimal global-knowledge policy and the swap-asap policy.

**Fig. 6:**
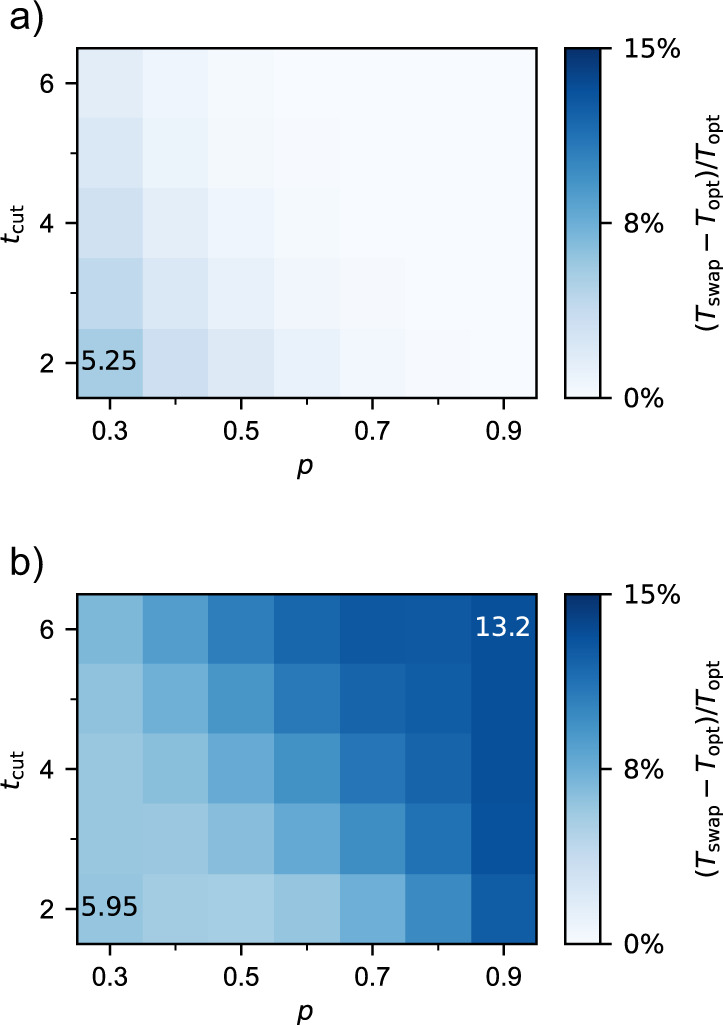
In a five-node chain, an optimal policy performs increasingly better than swap-asap for lower/higher values of *p* and *t*_cut_ when swaps are deterministic/probabilistic. Relative difference between the expected delivery times of an optimal policy, *T*_opt_, and the swap-asap policy, *T*_swap_, in a five-node chain, for different values of *p* and *t*_cut_. (**a**) Deterministic swaps (*p*_s_ = 1). (**b**) Probabilistic swaps (*p*_s_ = 0.5).

Figure 6 shows the relative difference between the expected delivery times of an optimal global-knowledge policy, *T*_opt_, and that of the swap-asap policy, *T*_swap_, in a five-node chain. Increasing values of (*T*_swap_ − *T*_opt_)/*T*_opt_ mean that the optimal policy is increasingly faster on average. Note that we restrict our analysis to the parameter regime *p* ≥ 0.3 and 2 ≤ *t*_cut_ ≤ 6 due to the very large computational cost of calculating the solution for smaller *p* and larger *t*_cut_ (for more details, see the Methods Section). Let us first focus on deterministic swaps (Fig. 6a). The advantage provided by an optimal policy increases for decreasing *p*. When *p* is small, links are more valuable since they are harder to generate. Therefore, it is convenient to avoid early swaps, as they effectively increase the ages of the links involved and make them expire earlier. When *t*_cut_ is small, a similar effect happens: all entangled links must be generated within a small time window and early swaps can make them expire too soon. For larger *t*_cut_, increasing the age of a link does not have a strong impact on the delivery time, since the time window is larger. Therefore, an optimal policy is increasingly better than swap-asap for decreasing *t*_cut_. The maximum difference between expected delivery times in the parameter region explored is 5.25%.

**Fig. 7:**
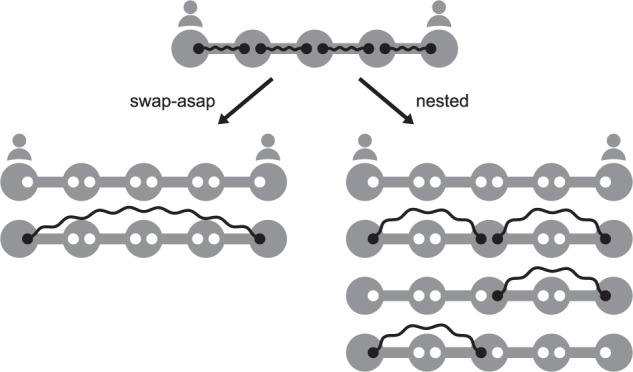
All possible transitions after performing a swap-asap action or a nested action in a full state, depending on which swaps succeed. In full states, every pair of neighbors shares an entangled link (solid black lines, with occupied qubits in black and free qubits in white). The swap-asap policy decides to swap all links, while the nested approach consists in swapping only at nodes 2 and 4. When swaps are probabilistic, the nested approach is generally optimal in terms of expected delivery time.

Interestingly, probabilistic swaps (Fig. 6b) yield an opposite behavior in the parameter region explored: optimal policies are increasingly better than swap-asap for increasing *p* and *t*_cut_ (except when *p* ≤ 0.4 and *t*_cut_ ≤ 3), and the relative difference in expected delivery time can be as large as 13.2% (achieved in a five-node chain with *p* = 0.9 and *t*_cut_ = 6). One reason for this may be the action that each policy decides to perform when the repeater chain is in a full state, which is a situation where each pair of neighboring nodes shares an entangled link (see state at the top of Fig. 7). When swaps are deterministic, the optimal policy chooses to swap all links in a full state, since end-to-end entanglement will always be achieved. However, when swaps are probabilistic, an optimal policy generally chooses to perform two separate swaps (see Fig. 7), similar to the nested purification scheme proposed in ref. ^27^. As an example, for *n* = 5, *p* = 0.9, *t*_cut_ = 2, and *p*_s_ = 0.5, the swap-asap policy yields an expected delivery time of *T* = 9.35. If, in full states, the swap at the third node is withheld, *T* drops to 8.34. The swap-asap policy is on average slower than this modified policy by 12.1%. The action chosen in full states has a stronger influence on *T* for increasing *p*. This is because full states are more frequent for large *p*: whenever a swap fails, a full state is soon recovered, since new entangled states are generated with high probability. As a consequence, an optimal policy is increasingly better than swap-asap for higher *p* when swaps are probabilistic. A similar effect happens for large *t*_cut_. Note however that the effect of the action chosen in full states is practically irrelevant in four-node chains (see Supplementary Note 3). Note also that the advantage of an optimal policy in terms of delivery time is not always monotonic in *p* and *t*_cut_ (see Supplementary Note 3).

**Fig. 8:**
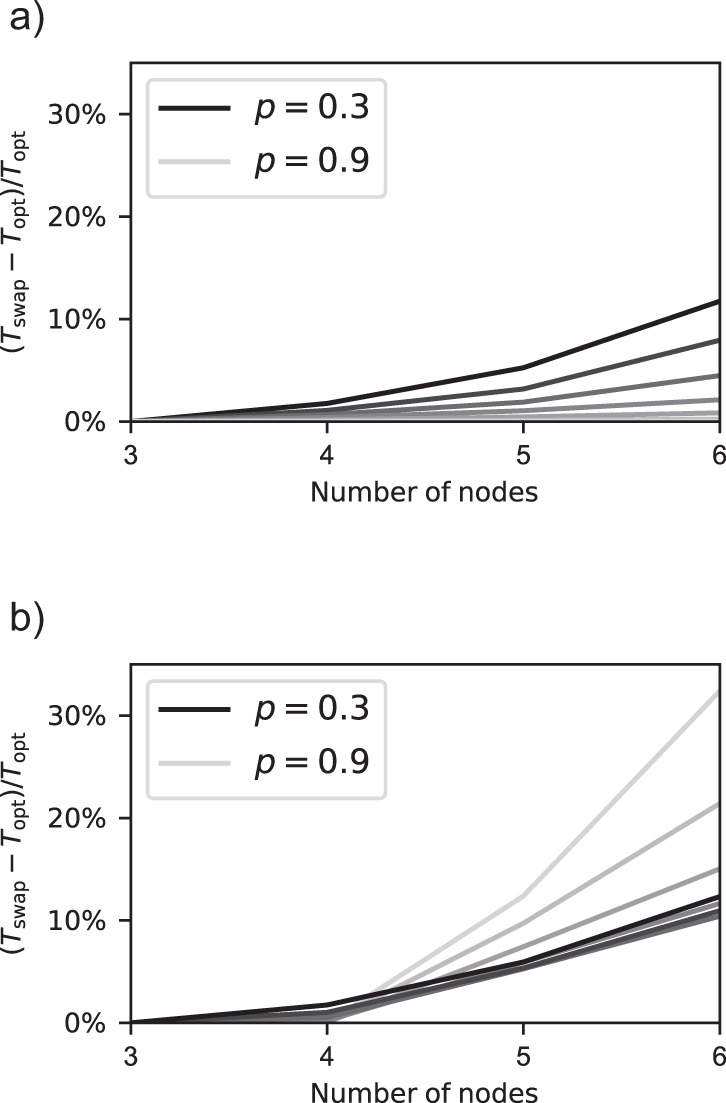
An optimal policy performs increasingly better than swap-asap in longer chains. Relative difference between the expected delivery times of an optimal policy, *T*_opt_, and the swap-asap policy, *T*_swap_, for *t*_cut_ = 2 and different values of *p*, as a function of the number of nodes *n*. Black lines correspond to *p* = 0.3, and the value of *p* increases in steps of 0.1 with increasing line transparency up to *p* = 0.9. (**a**) Deterministic swaps (*p*_s_ = 1). (**b**) Probabilistic swaps (*p*_s_ = 0.5).

Optimal policies are also increasingly faster than swap-asap for increasing *n*, as shown in Fig. 8. For example, for *p* = 0.3, *p*_s_ = 0.5, and *t*_cut_ = 2, the relative difference in expected delivery time is 1.7%, 5.9%, and 12.3%, for *n* = 4, 5, and 6, respectively. This is in line with the fact that, when the number of nodes grows, there are increasingly more states in which the optimal action to perform is a strict subset of all possible swaps, as shown in Supplementary Note 4. Note that, in three- and four-node chains, the relative difference in expected delivery time is generally below 1%.

## Discussion

Our work sheds light on how to distribute entanglement in quantum networks using a chain of intermediate repeaters with pre-configured cutoffs. We have shown that optimal global-knowledge policies can significantly outperform other policies, depending on the properties of the network. In particular, we have found and explained non-trivial examples in which performing swaps as soon as possible is far from optimal. We have also contributed a simple methodology to calculate optimal policies in repeater chains with cutoffs that can be extended to more realistic scenarios, e.g., asymmetric repeater chains, by modifying the transition probabilities of the MDP.

In this work, we have assumed that classical communication is instantaneous. Hence, our optimal policies may become sub-optimal in setups with non-negligible communication times, where decisions must be made using local information only. Nevertheless, our optimal policies still constitute a best-case policy against which to benchmark.

Note also that we have restricted our analysis to repeater chains with less than seven nodes. This is due to the exponentially large computational cost of solving the MDP for larger chains (see Supplementary Note 7 for further details). However, each entanglement swap decreases the fidelity of the entangled links. Hence, a large number of swaps limits the maximum end-to-end fidelity achievable, making chains with a very large number of nodes impractical. Therefore, we consider the analysis of short chains to be more relevant.

An interesting extension of this work would be to explore different cutoff policies. For example, one could allow the nodes to decide when to discard entangled links, or one could optimize simultaneously over the cutoff and the swapping policy. This may lead to improved optimal policies.

As a final remark, note that we have employed the expected delivery time as the single performance metric. In some cases, the expected value and the variance of the delivery time distribution are within the same order of magnitude (some examples are shown in Supplementary Note 5). Therefore, an interesting follow-up analysis would be to study the delivery time probability distribution instead of only the expected value. Additionally, we put fidelity aside by only requiring an end-to-end fidelity larger than some threshold value, via a constraint on the cutoff time. This constraint can be lifted to optimize the fidelity instead of the expected delivery time, or to formulate a multi-objective optimization problem to maximize fidelity while minimizing delivery time.

## Methods

### Finding optimal policies with a Markov decision process

We have formulated the problem of finding optimal entanglement distribution policies as an MDP where each state is a combination of existing entangled links and link ages. Let ***s*** be the state of the repeater chain at the beginning of a time slot. As previously explained, ***s*** can be described using the age of every entangled link. Mathematically, this means that ***s*** can be represented as a vector of size $$\left(\begin{array}{c}n\\ 2\end{array}\right)$$:

$${{{\boldsymbol{s}}}}=\left[{g}_{1}^{2},{g}_{1}^{3},\ldots ,{g}_{1}^{n};{g}_{2}^{3},\ldots ,{g}_{2}^{n};\ldots ;{g}_{n-1}^{n}\right],$$

where ${g}_{i}^{j}$ is the age of the entangled link between nodes *i* and *j* (if nodes *i* and *j* do not share an entangled link, then ${g}_{i}^{j}=-1$). In each time slot, the nodes must choose and perform an action *a*. Mathematically, *a* is a set containing the indices of the nodes that must perform swaps (if no swaps are performed, $a={{\emptyset}}$).

The state of the chain at the end of the time slot is ${{{{\boldsymbol{s}}}}}^{{\prime} }$. Since entanglement generation and swaps are probabilistic, the transition from ***s*** to ${{{{\boldsymbol{s}}}}}^{{\prime} }$ after performing *a* happens with some transition probability $P({{{{\boldsymbol{s}}}}}^{{\prime} }| {{{\boldsymbol{s}}}},a)$. A policy is a function *π* that indicates the action that must be performed at each state, i.e.,

$$\pi :{{{\boldsymbol{s}}}}\in {{{\mathcal{S}}}}\to \pi ({{{\boldsymbol{s}}}})\in {{{\mathcal{A}}}},$$

where ${{{\mathcal{S}}}}$ is the state space and ${{{\mathcal{A}}}}$ is the action space. W.l.o.g., we only consider deterministic policies, otherwise a policy would be a probability distribution instead of a function (see Supplementary Note 6 for further details).

Let us define ***s***_0_ as the state where no links are present and ${{{{\mathcal{S}}}}}_{{{{\rm{end}}}}}$ as the set of states with end-to-end entanglement, also called absorbing states. In general, the starting state is ***s***_0_, and the goal of the repeater chain is to transition to a state in ${{{{\mathcal{S}}}}}_{{{{\rm{end}}}}}$ in the fewest number of steps. When a state in ${{{{\mathcal{S}}}}}_{{{{\rm{end}}}}}$ is reached, the process stops. Let us define the expected delivery time from state ***s*** when following policy *π*, *T*_*π*_(***s***), as the expected number of steps required to reach an absorbing state when starting from state ***s***. The expected delivery time is also called hitting time in the context of Markov chains (see Chapter 9 from ref. ^40^). A policy *π* is better than or equal to a policy ${\pi }^{{\prime} }$ if ${T}_{\pi }({{{\boldsymbol{s}}}})\le {T}_{{\pi }^{{\prime} }}({{{\boldsymbol{s}}}})$, $\forall {{{\boldsymbol{s}}}}\in {{{\mathcal{S}}}}$. An optimal policy π^*^ is one that is better than or equal to all other policies. In other words, an optimal policy is one that minimizes the expected delivery time from all states. One can show that there exists at least one optimal policy in an MDP with a finite and countable set of states (see Section 2.3 from ref. ^41^). To find such an optimal policy, we employ the following set of equations, which are derived in Supplementary Note 6:

5 $${T}_{\pi }({{{\boldsymbol{s}}}})=1+\mathop{\sum}\limits_{{{{{\boldsymbol{s}}}}}^{{\prime} }\in {{{\mathcal{S}}}}}P({{{{\boldsymbol{s}}}}}^{{\prime} }| {{{\boldsymbol{s}}}},\pi )\cdot {T}_{\pi }({{{{\boldsymbol{s}}}}}^{{\prime} }),\forall {{{\boldsymbol{s}}}}\in {{{\mathcal{S}}}},$$

where ${{{\mathcal{S}}}}$ is the state space and $P({{{{\boldsymbol{s}}}}}^{{\prime} }| {{{\boldsymbol{s}}}},\pi )$ is the probability of transition from state ***s*** to state ${{{{\boldsymbol{s}}}}}^{{\prime} }$ when following policy *π*. Equations (5) are a particular case of what is generally known in the literature as the Bellman equations.

An optimal policy can be found by minimizing *T*_*π*_(***s***), $\forall {{{\boldsymbol{s}}}}\in {{{\mathcal{S}}}}$, using (5). To solve this optimization problem, we used value iteration and policy iteration, which are two different iterative methods whose solution converges to the optimal policy (both methods provided the same results). For more details, see Supplementary Note 7, and for a general reference on value and policy iteration, see Chapter 4 from ref. ^39^.

We provide an example of how to calculate the transition probabilities $P({{{{\boldsymbol{s}}}}}^{{\prime} }| {{{\boldsymbol{s}}}},\pi )$ analytically in Supplementary Note 8, although this is generally impractical, since the size of the state space grows at least exponentially with *n* and polynomially with *t*_cut_ (as shown in Supplementary Note 9, $| {{{\mathcal{S}}}}| =\Omega ({({t}_{{{{\rm{cut}}}}})}^{n-2})$). Lastly, in Supplementary Note 10 we discuss how to simplify the calculation of transition probabilities.

As a validation check, we also implemented a Monte Carlo simulation that can run our optimal policies, providing the same expected delivery time that we obtained from solving the MDP.

### Supplementary information

Supplementary Notes

## Data Availability

The data shown in this paper can be found in ref. ^42^.

## Code availability

Our code can be found in the following GitHub repository: https://github.com/AlvaroGI/optimal-homogeneous-chain.

## References

[1] Ekert AK (1991). Quantum cryptography based on Bell’s theorem. Phys. Rev. Lett..

[2] Bennett CH, Brassard G, Mermin ND (1992). Quantum cryptography without Bell’s theorem. Phys. Rev. Lett..

[3] Ben-Or, M., Crépeau, C., Gottesman, D., Hassidim, A. & Smith, A. Secure multiparty quantum computation with (only) a strict honest majority. In *2006 47th Annual IEEE Symposium on Foundations of Computer Science (FOCS’06)*, 249–260 (IEEE, 2006).

[4] Pirker A, Wallnöfer J, Dür W (2018). Modular architectures for quantum networks. New J. Phys..

[5] Kruszynska C, Anders S, Dür W, Briegel HJ (2006). Quantum communication cost of preparing multipartite entanglement. Phys. Rev. A.

[6] Bugalho L, Coutinho BC, Omar Y (2021). Distributing multipartite entanglement over noisy quantum networks. Quantum.

[7] Yoshino K, Ochi T, Fujiwara M, Sasaki M, Tajima A (2013). Maintenance-free operation of WDM quantum key distribution system through a field fiber over 30 days. Opt. Express.

[8] Stephenson LJ (2020). High-rate, high-fidelity entanglement of qubits across an elementary quantum network. Phys. Rev. Lett..

[9] Ursin R (2007). Entanglement-based quantum communication over 144 km. Nat. Phys..

[10] Sidhu JS (2021). Advances in space quantum communications. IET Quantum Comm..

[11] Duan LM, Lukin MD, Cirac JI, Zoller P (2001). Long-distance quantum communication with atomic ensembles and linear optics. Nature.

[12] Sangouard N, Simon C, De Riedmatten H, Gisin N (2011). Quantum repeaters based on atomic ensembles and linear optics. Rev. Mod. Phys..

[13] Bernien H (2013). Heralded entanglement between solid-state qubits separated by three metres. Nature.

[14] Hensen B (2015). Loophole-free bell inequality violation using electron spins separated by 1.3 kilometres. Nature.

[15] Humphreys PC (2018). Deterministic delivery of remote entanglement on a quantum network. Nature.

[16] Rozpędek F (2019). Near-term quantum-repeater experiments with nitrogen-vacancy centers: overcoming the limitations of direct transmission. Phys. Rev. A.

[17] Pompili M (2021). Realization of a multinode quantum network of remote solid-state qubits. Science.

[18] Moehring DL (2007). Entanglement of single-atom quantum bits at a distance. Nature.

[19] Slodička L (2013). Atom-atom entanglement by single-photon detection. Phys. Rev. Lett..

[20] Barrett SD, Kok P (2005). Efficient high-fidelity quantum computation using matter qubits and linear optics. Phys. Rev. A.

[21] Żukowski M, Zeilinger A, Horne MA, Ekert AK (1993). "Event-ready-detectors” Bell experiment via entanglement swapping. Phys. Rev. Lett..

[22] Collins OA, Jenkins SD, Kuzmich A, Kennedy TAB (2007). Multiplexed memory-insensitive quantum repeaters. Phys. Rev. Lett..

[23] Rozpędek F (2018). Parameter regimes for a single sequential quantum repeater. Quantum Sci. Technol..

[24] Khatri S, Matyas CT, Siddiqui AU, Dowling JP (2019). Practical figures of merit and thresholds for entanglement distribution in quantum networks. Phys. Rev. Res..

[25] Li, B., Coopmans, T. & Elkouss, D. Efficient optimization of cut-offs in quantum repeater chains. In *2020 IEEE International Conference on Quantum Computing and Engineering (QCE)*, 158–168 (IEEE, 2020).

[26] Coopmans T, Brand S, Elkouss D (2022). Improved analytical bounds on delivery times of long-distance entanglement. Phys. Rev. A.

[27] Briegel HJ, Dür W, Cirac JI, Zoller P (1998). Quantum repeaters: the role of imperfect local operations in quantum communication. Phys. Rev. Lett..

[28] Jiang L, Taylor JM, Khaneja N, Lukin MD (2007). Optimal approach to quantum communication using dynamic programming. Proc. Natl Acad. Sci. USA.

[29] Shchukin E, Schmidt F, van Loock P (2019). Waiting time in quantum repeaters with probabilistic entanglement swapping. Phys. Rev. A.

[30] Vardoyan G, Guha S, Nain P, Towsley D (2021). On the capacity region of bipartite and tripartite entanglement switching. ACM SIGMETRICS Performance Evaluation Rev..

[31] Vardoyan G, Guha S, Nain P, Towsley D (2021). On the stochastic analysis of a quantum entanglement distribution switch. IEEE Trans. Quant. Eng..

[32] Khatri S (2022). On the design and analysis of near-term quantum network protocols using Markov decision processes. AVS Quantum Sci..

[33] Shchukin E, van Loock P (2022). Optimal entanglement swapping in quantum repeaters. Phys. Rev. Lett..

[34] Werner RF (1989). Quantum states with Einstein-Podolsky-Rosen correlations admitting a hidden-variable model. Phys. Rev. A.

[35] Dür W, Hein M, Cirac JI, Briegel HJ (2005). Standard forms of noisy quantum operations via depolarization. Phys. Rev. A.

[36] Munro WJ, Azuma K, Tamaki K, Nemoto K (2015). Inside quantum repeaters. IEEE J. Selected Top. Quant. Electron..

[37] Chirolli L, Burkard G (2008). Decoherence in solid-state qubits. Adv. Phys..

[38] Calsamiglia J, Lütkenhaus N (2001). Maximum efficiency of a linear-optical Bell-state analyzer. Appl. Phys. B.

[39] Sutton RS, Barto AG (2018). Reinforcement Learning: an Introduction.

[40] Van Mieghem P (2014). Performance Analysis of Complex Networks and Systems.

[41] Szepesvári C (2010). Algorithms for reinforcement learning. Synthesis lectures on Artificial Intelligence and Machine Learning.

[42] Iñesta, Á. G., Vardoyan, G., Scavuzzo, L. & Wehner, S. Data for ’Optimal entanglement distribution policies in homogeneous repeater chains with cutoffs’. *4TU.ResearchData*, 10.4121/20402037.v1 (2022). 10.1038/s41534-023-00713-9 PMC11041801 38665258

