# Supplementary material for: Optimal entanglement distribution policies in homogeneous repeater chains with cutoffs
Source: npj Quantum Inf. 2023 May 6;9(1):46. doi: 10.1038/s41534-023-00713-9 (PMC11041801; doi:10.1038/s41534-023-00713-9)
Supplement: Supplementary file 1 — Supplementary Notes [file 41534_2023_713_MOESM1_ESM.pdf]

Optimal entanglement distribution policies in homogeneous repeater chains with cutoffs: Supplementary Notes

Álvaro G. Iñesta,[1,2,3,*] Gayane Vardoyan,[1,2,†] Lara Scavuzzo,[2] and Stephanie Wehner[1,2,3]
[1] *QuTech, Delft University of Technology, Lorentzweg 1, 2628 CJ Delft, The Netherlands*
[2] *EEMCS, Delft University of Technology, Mekelweg 4, 2628 CD Delft, The Netherlands*
[3] *Kavli Institute of Nanoscience, Delft University of Technology, Lorentzweg 1, 2628 CJ Delft, The Netherlands*
(Dated: April 12, 2023)

Supplementary Note 1. DEPOLARIZATION OF WERNER STATES

In this Supplementary Note we show that the fidelity of a Werner state in which each qubit independently experiences a depolarizing process evolves as

$$F(t) = \frac{1}{4} + \left(F(t-\Delta t) - \frac{1}{4}\right)\mathrm{e}^{-\frac{\Delta t}{\tau}},$$

where t is the time, Δt is an arbitrary interval of time, and τ is a parameter that characterizes the exponential decay in fidelity of the whole entangled state due to the qubits being stored in noisy memories. Note that we assume independent noise on each qubit since, in our problem, they are stored in different nodes of the repeater chain.

The depolarizing channel [1, 2] is defined as

$$\mathcal{E}_i : \quad \rho_i \quad \rightarrow \quad p\rho_i + (1-p)\frac{\mathbb{I}_2}{2}, \tag{1}$$

where ρ_i is a single-qubit state, $0 \leq p \leq 1$ (this p is not to be confused with the entanglement generation probability used in the main text of this paper), and $\mathbb{I}_d$ is the d-dimensional identity. Let us assume that each qubit independenlty experiences a depolarizing channel while stored in memory for a finite time t_{dep}. During an interval of time t_{dep}, a Werner state ρ with fidelity F is therefore mapped to $(\mathcal{E}_1 \otimes \mathcal{E}_2)(\rho)$, where $\mathcal{E}_i$ is a depolarizing channel acting on the i-th qubit. Let us calculate this output state explicitly:

$$\begin{aligned}
(\mathcal{E}_1 \otimes \mathcal{E}_2)(\rho) &= p^2\rho + p(1-p)\,\mathrm{Tr}_2(\rho) \otimes \frac{\mathbb{I}_2}{2} + p(1-p)\frac{\mathbb{I}_2}{2} \otimes \mathrm{Tr}_1(\rho) + (1-p)^2\frac{\mathbb{I}_4}{4} \\
&\overset{a}{=} p^2\rho + p(1-p)\frac{\mathbb{I}_2}{2} \otimes \frac{\mathbb{I}_2}{2} + p(1-p)\frac{\mathbb{I}_2}{2} \otimes \frac{\mathbb{I}_2}{2} + (1-p)^2\frac{\mathbb{I}_4}{4} \\
&= p^2\rho + \left(2p(1-p) + (1-p)^2\right)\frac{\mathbb{I}_4}{4} \\
&\overset{b}{=} p^2\frac{4F-1}{3}\left|\phi^+\middle\rangle\middle\langle\phi^+\right| + p^2\frac{1-F}{3}\mathbb{I}_4 + \left(2p(1-p) + (1-p)^2\right)\frac{\mathbb{I}_4}{4} \\
&\overset{c}{=} \frac{4F'-1}{3}\left|\phi^+\middle\rangle\middle\langle\phi^+\right| + \frac{1-F'}{3}\mathbb{I}_4,
\end{aligned} \tag{2}$$

with the following steps:

a. We use the fact that the partial trace of a maximally entangled state is a maximally mixed state. As a consequence, $\mathrm{Tr}_i(\rho) = \frac{\mathbb{I}_2}{2}$, for any Werner state ρ.

b. We use the definition of Werner state: $\rho = \frac{4F-1}{3}\left|\phi^+\middle\rangle\middle\langle\phi^+\right| + \frac{1-F}{3}\mathbb{I}_4$.

c. We define $F' = \frac{1}{4} + p^2(F - \frac{1}{4})$.

The output state $(\mathcal{E}_1 \otimes \mathcal{E}_2)(\rho)$ is a Werner state with fidelity F'. Then, the application of n successive transformations $\mathcal{E}_1 \otimes \mathcal{E}_2$ produces a Werner state with fidelity

$$F^{(n)} = \frac{1}{4} + p^{2n}\left(F - \frac{1}{4}\right). \tag{3}$$

* a.gomezinesta@tudelft.nl
† g.s.vardoyan@tudelft.nl

This can be shown by induction as follows. The base case is proven in (2): $F^{(1)} = \frac{1}{4} + p^2(F - \frac{1}{4})$. Next, if we assume that (3) is true for $n = k$, we can show that it also holds for $n = k+1$:

$$F^{(k+1)} = \frac{1}{4} + p^2\left(F^{(k)} - \frac{1}{4}\right) = \frac{1}{4} + p^2\left(\frac{1}{4} + p^{2k}\left(F - \frac{1}{4}\right) - \frac{1}{4}\right) = \frac{1}{4} + p^{2(k+1)}\left(F - \frac{1}{4}\right),$$

where we have used (2) in the first step.

The total time required for these operations is $\Delta t = n t_{\text{dep}}$. Therefore, if the fidelity of the state at time $t - \Delta t$ was $F(t - \Delta t)$, the fidelity at t is given by

$$F(t) = \frac{1}{4} + p^{2\Delta t/t_{\text{dep}}}\left(F(t - \Delta t) - \frac{1}{4}\right). \tag{4}$$

Finally, we map $p \in [0,1]$ to a new parameter $\tau \in [0, +\infty)$ as $p^2 \equiv \mathrm{e}^{-t_{\text{dep}}/\tau}$. Then, we obtain

$$F(t) = \frac{1}{4} + \left(F(t - \Delta t) - \frac{1}{4}\right)\mathrm{e}^{-\frac{\Delta t}{\tau}}. \tag{5}$$

Supplementary Note 2. RELATION BETWEEN CUTOFF AND THRESHOLD FIDELITY

In the design of a quantum repeater chain, we must select a cutoff time t_{cut} such that the fidelity of any end-to-end entangled link is larger than some threshold F_{min}. We show that this requirement is always satisfied when the cutoff time meets the following condition:

$$t_{\text{cut}} \leq -\tau \ln\left(\frac{3}{4F_{\text{new}} - 1}\left(\frac{4F_{\text{min}} - 1}{3}\right)^{\frac{1}{n-1}}\right), \tag{6}$$

where τ is a parameter that characterizes the exponential decay in fidelity of the whole entangled state due to the qubits being stored in noisy memories, F_{new} is the fidelity of newly generated entangled links, F_{min} is the minimum desired end-to-end fidelity, and n is the number of nodes in the chain.

First, we analyze the impact of a late entanglement swap on the fidelity of the output state. As shown in Supplementary Note 1, the fidelity of a Werner state that experiences depolarizing noise independently on each qubit decays as

$$F(t) = \frac{1}{4} + \left(F(t - \Delta t) - \frac{1}{4}\right)\mathrm{e}^{-\Delta t/\tau}, \tag{7}$$

over an interval of time time Δt. When two Werner states are used as input in an entanglement swap, the output state is a Werner state with fidelity

$$F_{\text{swap}}(F_1, F_2) = F_1 \cdot F_2 + \frac{(1 - F_1)\cdot(1 - F_2)}{3}, \tag{8}$$

where F_1 and F_2 are the fidelities of the input states [3].

Let us consider two Werner states with initial fidelities $F_1(t_0)$ and $F_2(t_0)$, respectively. On the one hand, if we perform a swap and then wait for some time t_{wait}, the final state is a Werner state with fidelity

$$F_{\text{swap-wait}}(t_0 + t_{\text{wait}}) = \frac{1}{4} + \left(F_1(t_0)F_2(t_0) - \frac{1}{4} + \frac{\big(1 - F_1(t_0)\big)\big(1 - F_2(t_0)\big)}{3}\right)\mathrm{e}^{-t_{\text{wait}}/\tau}, \tag{9}$$

which can be obtained by applying (8) first and (7) next. On the other hand, if we wait for some time t_{wait} and then perform the swap, we obtain a Werner state with fidelity

$$\begin{aligned} F_{\text{wait-swap}}(t_0 + t_{\text{wait}}) &= F_1(t_0 + t_{\text{wait}})F_2(t_0 + t_{\text{wait}}) + \frac{\big(1 - F_1(t_0 + t_{\text{wait}})\big)\big(1 - F_2(t_0 + t_{\text{wait}})\big)}{3} \\ &= \frac{1}{4} + \left(F_1(t_0)F_2(t_0) - \frac{1}{4} + \frac{\big(1 - F_1(t_0)\big)\big(1 - F_2(t_0)\big)}{3}\right)\mathrm{e}^{-2t_{\text{wait}}/\tau}, \end{aligned} \tag{10}$$

where we have used (7) in the second step and performed some basic algebra. Note that the factor that multiplies the exponential in (9) and (10) is nonnegative as long as $F_1(t_0), F_2(t_0) \geq \frac{1}{4}$ – if the initial fidelity is $\frac{1}{4}$, the initial state is a maximally mixed state.

By comparing (9) and (10), we find that an entangled link with larger fidelity is obtained if we first perform an entanglement swap and then wait for time t_{wait} rather than if we wait for time t_{wait} and then perform the swap, since

$$F_{\text{swap-wait}}(t_0 + t_{\text{wait}}) > F_{\text{wait-swap}}(t_0 + t_{\text{wait}}), \quad \forall t_{\text{wait}} > 0. \tag{11}$$

Let us now consider a sequence of m entangled links that can be fused into a single long link after performing $m-1$ swaps. Each of the initial links has fidelity F_i, $i = 1, ..., m$. We want to calculate the final fidelity, assuming that all swaps are successful. For this, it is convenient to define a Werner state in terms of the Werner parameter x:

$$\rho = x \left|\phi^+\middle\rangle\!\middle\langle\phi^+\right| + \frac{1-x}{4}\mathbb{I}_4, \tag{12}$$

where $|\phi^+\rangle = \frac{|00\rangle+|11\rangle}{\sqrt{2}}$ is a Bell state, and $\mathbb{I}_d$ is the d-dimensional identity. The Werner parameter x is defined in terms of the fidelity as $x = \frac{4F-1}{3}$. Equation (8) can be written in terms of the Werner parameter of each state:

$$x_{\text{swap}}(x_1, x_2) = x_1 x_2, \tag{13}$$

where x_{swap} is the Werner parameter of the output state after swapping two Werner states with Werner parameters x_1 and x_2. If we apply Equation (13) repeatedly to our sequence of m entangled links, assuming that all swaps happen simultaneously (i.e., with no decoherence happening in between swaps), we obtain a final state with Werner parameter

$$x_{\text{final}} = x_1 x_2 \dots x_m = \prod_{i=1}^{m} \frac{4F_i - 1}{3}. \tag{14}$$

Then, the final fidelity is given by

$$F_{\text{final}} = \frac{3x_{\text{final}} + 1}{4} = \frac{1}{4} + \frac{3}{4}\prod_{i=1}^{m} \frac{4F_i - 1}{3}. \tag{15}$$

A similar result was derived in [4], although assuming $F_i = F$, $\forall i$.

We are now ready to find a relationship between the cutoff time and the minimum fidelity in an n-node quantum repeater chain with cutoff time t_{cut}. For this, we need to identify the sequence of events that produces the end-to-end link with the lowest fidelity. First, note that all the entangled links that eventually form a single end-to-end link are created within a window of t_{cut} time slots. According to (11), delaying entanglement swaps has a negative impact on the final fidelity. Therefore, the sequence of events that produces end-to-end entanglement with the lowest fidelity must be one where all swaps are performed at the end of the time window, i.e., all swaps are performed when the oldest link reaches the cutoff time, just before it expires. If any of those swaps were performed earlier, the final fidelity would be larger. Such a sequence of events produces the lowest end-to-end fidelity when all the links are as old as possible, i.e., when their age is t_{cut}. If any of the links were younger, the end-to-end fidelity would be larger. Hence, the lowest end-to-end fidelity is achieved when all the links are generated simultaneously and all the swaps are performed when those links are t_{cut} time slots old. In this case, the fidelity of each link before swapping is given by (7):

$$F_{\text{old}} = \frac{1}{4} + \left(F_{\text{new}} - \frac{1}{4}\right) e^{-\frac{t_{\text{cut}}}{\tau}}, \tag{16}$$

where F_{new} is the fidelity of newly generated elementary links. The final fidelity after swapping all the links can be calculated using (15):

$$F_{\text{worst}} = \frac{1}{4} \cdot \left[1 + \frac{(4F_{\text{old}} - 1)^{n-1}}{3^{n-2}}\right]. \tag{17}$$

Finally, we impose that the worst-case end-to-end fidelity must be larger than the desired minimum fidelity F_{min}: $F_{\text{worst}} \geq F_{\text{min}}$. Solving for t_{cut} yields an explicit condition for the cutoff time:

$$t_{\text{cut}} \leq -\tau \ln\left(\frac{3}{4F_{\text{new}} - 1}\left(\frac{4F_{\text{min}} - 1}{3}\right)^{\frac{1}{n-1}}\right). \tag{18}$$

When this condition is satisfied, every sequence of events will lead to a large enough fidelity. Consequently, any policy that we implement on the repeater chain will also deliver entanglement with a large enough fidelity.

Supplementary Note 3. FURTHER COMMENTS ON THE EXPECTED DELIVERY TIME OF OPTIMAL POLICIES

Here we provide the expected delivery time of optimal policies in three- and four-node repeater chains. Then, we compare optimal policies to the swap-asap policy in a four-node chain. We also show that, in longer chains, the relative difference in expected delivery time is not always monotonic with the probability of successful entanglement generation p.

Supplementary Figure 1 shows the expected delivery time of an optimal policy, $T_{\rm opt}$, in three- and four-node chains, versus p for different values of $p_{\rm s}$ and $t_{\rm cut}$. The relation between $T_{\rm opt}$ and the rest of the variables is similar to that of the five-node chain discussed in the main text. When p is small, more entanglement generation attempts are required to succeed, yielding a larger $T_{\rm opt}$. Decreasing $p_{\rm s}$ also increases $T_{\rm opt}$, since more attempts at entanglement swapping are required on average. When $t_{\rm cut}$ is small, all entangled states must be generated within a small time window and therefore $T_{\rm opt}$ is also larger.

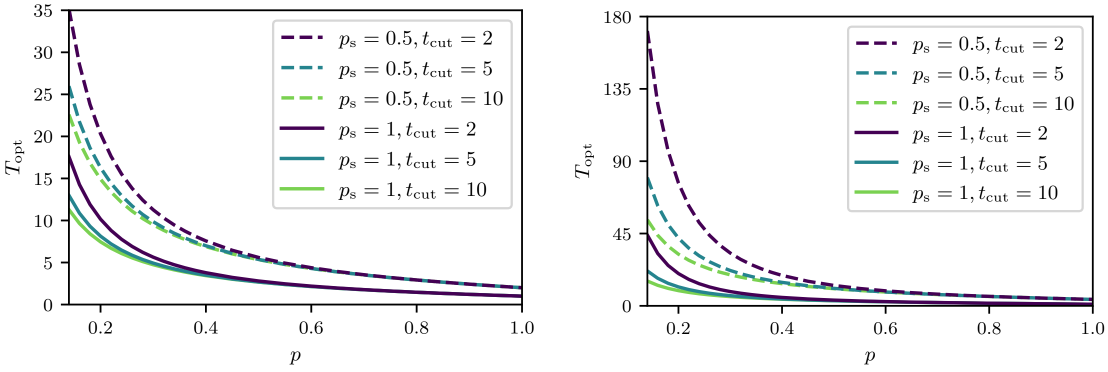

(a) Three-node chain. **(b)** Four-node chain.

Supplementary Figure 1. The expected delivery time increases with lower p, $p_{\rm s}$, and $t_{\rm cut}$. Expected delivery time of an optimal policy, $T_{\rm opt}$, versus p for (a) $n = 3$ and (b) $n = 4$ and different values of cutoff ($t_{\rm cut} = 2, 5, 10$). Solid lines correspond to deterministic swaps ($p_{\rm s} = 1$) and dashed lines correspond to probabilistic swaps with $p_{\rm s} = 0.5$.

In three-node chains, the swap-asap policy is always optimal since there is no reason to wait after both links have been generated. As the number of nodes increases, policies have more degrees of freedom that can be adjusted to get an improvement over the swap-asap policy. Supplementary Figure 2 shows the advantage in expected delivery time of an optimal policy versus the swap-asap policy in four-node chains. The swap-asap policy is no longer optimal, as it was in three-node chains, although the largest advantage observed is below 2%, meaning that the swap-asap policy is still close to optimal. The advantage over swap-asap increases up to 30% in five- and six-node chains, as shown in Supplementary Figure 3 and in Figure 8 from the main text.

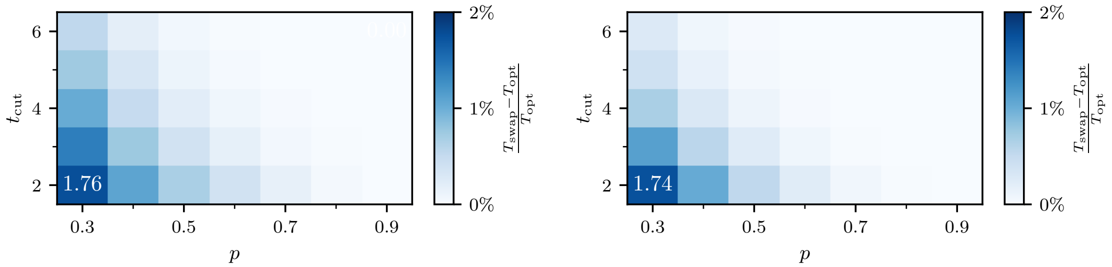

(a) Deterministic swaps ($p_{\rm s} = 1$). **(b)** Probabilistic swaps ($p_{\rm s} = 0.5$).

Supplementary Figure 2. The swap-asap policy is close to optimal in four-node chains. Relative difference between the expected delivery times of an optimal policy, $T_{\rm opt}$, and the swap-asap policy, $T_{\rm swap}$, in a four-node chain, for different values of p and $t_{\rm cut}$.

Supplementary Figure 3 shows the advantage of an optimal policy over swap-asap in terms of expected delivery time, versus p and for different number of nodes. When swaps are deterministic, the advantage is larger for smaller p. The reason is that links are harder to generate as p approaches zero, and therefore a fine-tuned policy that makes better use of those scarce resources is expected to be increasingly better than a greedy policy like swap-asap. When swaps are probabilistic and $n > 4$, the advantage is not monotonic in p anymore, as can clearly be seen for $n = 6$, $t_{\rm cut} = 2$, and $p_{\rm s} = 0.5$. On the

one hand, the advantage increases when p approaches zero, due to swap-asap making an inefficient use of the links, which become a scarce resource. On the other hand, when p approaches one, the advantage also increases, due to the effect of full states, as discussed in the main text.

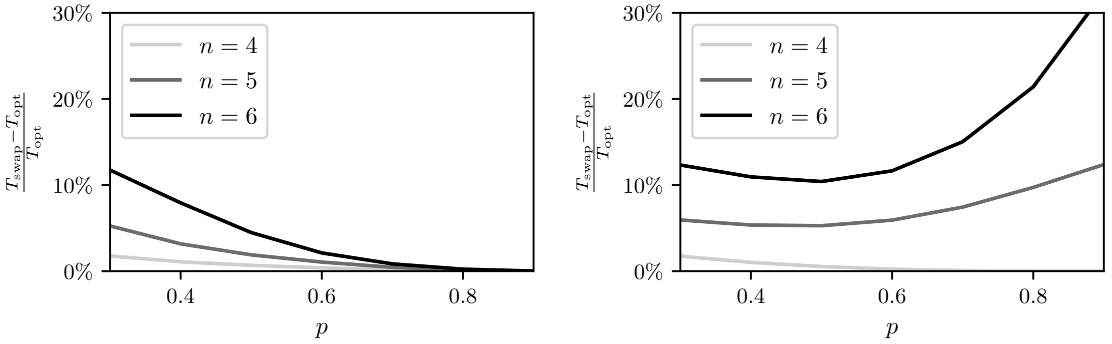

(a) Deterministic swaps ($p_{\mathrm{s}} = 1$).

(b) Probabilistic swaps ($p_{\mathrm{s}} = 0.5$).

Supplementary Figure 3. The advantage provided by an optimal policy over swap-asap is not always monotonic with p. Relative difference between the expected delivery times of an optimal policy, T_{opt}, and the swap-asap policy, T_{swap}, in an n-node chain with $t_{\mathrm{cut}} = 2$, for different values of n and p.

Supplementary Note 4. ANALYSIS OF THE ACTIONS OF OPTIMAL POLICIES

Supplementary Figure 4 shows the percentage of states in which an optimal policy decides to perform all possible swaps (acting as the swap-asap policy) or not perform any swap at all, in a five-node repeater chain. For this analysis, we only consider states in which at least one swap can be performed. Although there seem to be some clear trends, these results cannot be used to determine how close the swap-asap policy is to being optimal, since:

(i) We found one of the possibly many optimal policies, so the swap-asap policy may be closer to a different optimal policy. This also explains why the plots in Supplementary Figure 4 are not monotonic.

(ii) Even if there is only one state in which two policies differ, this state could have a large impact on the expected delivery time, as explained in the example of full states in the main text.

In Supplementary Figure 5 we plot the same quantities for increasing number of nodes. The percentage of states in which the optimal policy decides to perform all possible swaps, acting as the swap-asap policy, decreases with increasing n. This agrees with the fact that the advantage provided by an optimal policy in terms of expected delivery time over the swap-asap policy increases with increasing n, as shown in the main text. However, the data from Supplementary Figure 5 alone should not be used to draw any conclusions, since arguments (i) and (ii) also apply to these plots.

Supplementary Note 5. DELIVERY TIME DISTRIBUTION

Here, we show two examples of repeater chains in which the entanglement delivery time distribution is heavy-tailed. Supplementary Figure 6 shows the delivery time distribution in a five-node chain with $p_{\mathrm{s}} = 0.5$, $t_{\mathrm{cut}} = 2$, and $p = 0.5$ (Supplementary Figure 6a) or $p = 0.9$ (Supplementary Figure 6b). The results shown here have been calculated by repeatedly simulating the optimal policy in a repeater chain (source code available at https://github.com/AlvaroGI/optimal-homogeneous-chain). As shown in the figure, the distribution is heavy-tailed for some combinations of parameters. In those cases, the average value does not provide an accurate description of the whole distribution.

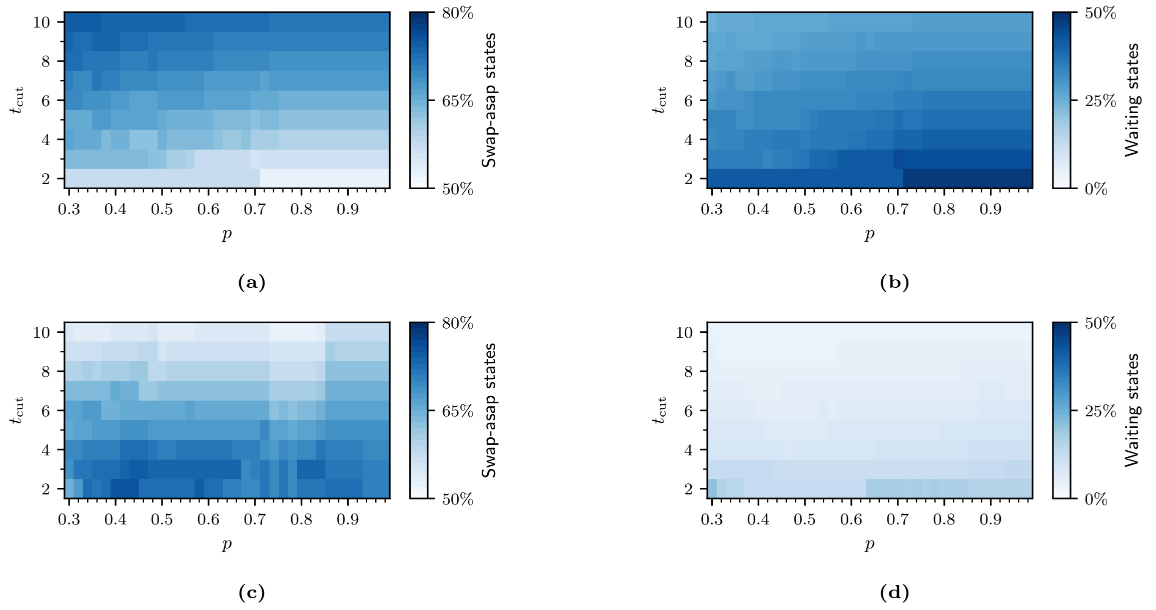

Supplementary Figure 4. An optimal policy acts as the swap-asap policy in a large number of states. Percentage of states in which the optimal policy found by our solver decides to (a,c) perform all possible swaps or (b,d) not perform any swap, in a five-node repeater chain with (a-b) $p_{\mathrm{s}} = 1$ or (c-d) $p_{\mathrm{s}} = 0.5$. We only consider states in which at least one swap can be performed.

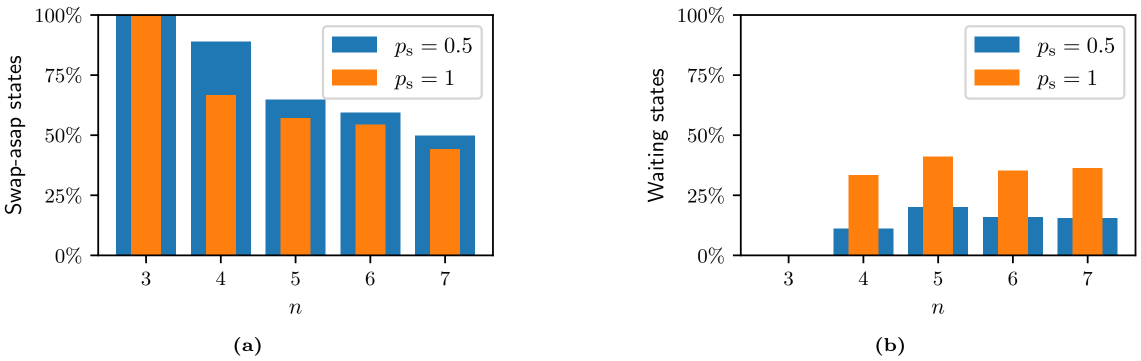

Supplementary Figure 5. The percentage of states in which the optimal policy acts as the swap-asap policy decreases in longer chains. Percentage of states in which the optimal policy found by our solver decides to (a) perform all possible swaps or (b) not perform any swap, in a repeater chain with $p = 0.3$ and $t_{\mathrm{cut}} = 2$. We only consider states in which at least one swap can be performed.

Supplementary Note 6. EXPECTED TIME TO REACH AN ABSORBING STATE

In this Supplementary Note, we show that the expected time required to reach an absorbing state in a discrete Markov decision process (MDP) starting from state $\boldsymbol{s}$ and following policy π, $T_\pi(\boldsymbol{s})$, satisfies

$$T_\pi(\boldsymbol{s}) = 1 + \sum_{\boldsymbol{s}' \in \mathcal{S}} P(\boldsymbol{s}'|\boldsymbol{s}, \pi) \cdot T_\pi(\boldsymbol{s}'),$$

where $\mathcal{S}$ is the state space and $P(\boldsymbol{s}'|\boldsymbol{s}, \pi)$ is the probability of transition from state $\boldsymbol{s}$ to state $\boldsymbol{s}'$ when following policy π. We also discuss the difference between deterministic and stochastic policies.

Let $t_\pi(\boldsymbol{s})$ be the time required to reach an absorbing state starting from state $\boldsymbol{s}$ in one realization of the process, and let

$$T_\pi(\boldsymbol{s}) \equiv \mathbb{E}[t_\pi(\boldsymbol{s})] = \sum_{m=0}^{\infty} m \Pr[t_\pi(\boldsymbol{s}) = m] \tag{19}$$

be its expected value. The time required to reach an absorbing state starting from $\boldsymbol{s}$ can be calculated as the time required to go from state $\boldsymbol{s}$ to any state $\boldsymbol{s}'$ plus the time required to go from $\boldsymbol{s}'$ to an absorbing state. Since the Markov chain is

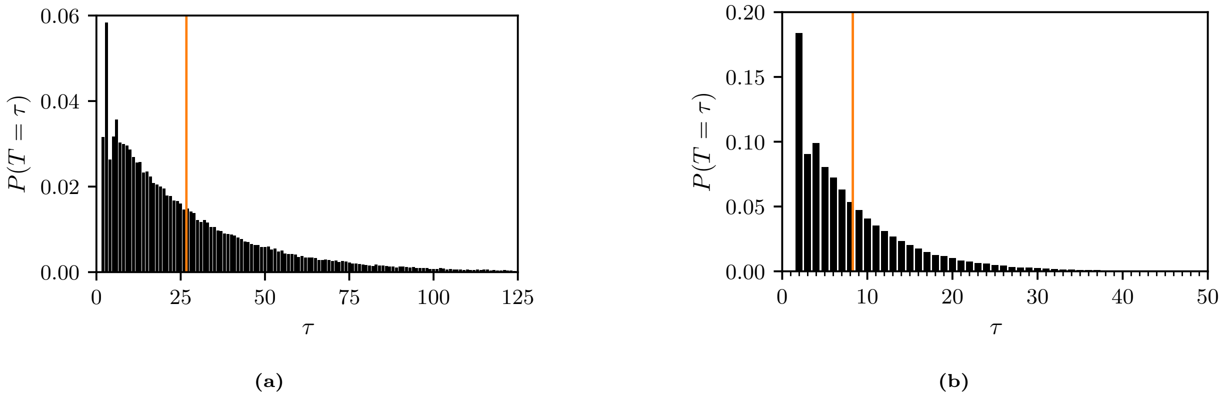

Supplementary Figure 6. The delivery time distribution can be heavy-tailed. Delivery time distribution after simulating an optimal policy in a five-node repeater chain with $p_{\mathrm{s}} = 0.5$, $t_{\mathrm{cut}} = 2$, and (a) $p = 0.5$ or (b) $p = 0.9$. The number of samples is 10^5. Solid orange lines correspond to the expected delivery time of the optimal policy.

discrete, we can write this as

$$\Pr[t_\pi(\boldsymbol{s}) = m] = \sum_{\boldsymbol{s}'\in\mathcal{S}} P(\boldsymbol{s}'|\boldsymbol{s},\pi)\Pr[t_\pi(\boldsymbol{s}') = m-1]. \tag{20}$$

The recursive relation for $T_\pi(\boldsymbol{s})$ can be derived as follows:

$$\begin{aligned}
T_\pi(\boldsymbol{s}) &\overset{a}{=} \sum_{m=0}^{\infty} m\Pr[t_\pi(\boldsymbol{s}) = m] && (21)\\
&\overset{b}{=} \sum_{m=0}^{\infty} m \sum_{\boldsymbol{s}'\in\mathcal{S}} P(\boldsymbol{s}'|\boldsymbol{s},\pi)\Pr[t_\pi(\boldsymbol{s}') = m-1] && (22)\\
&= \sum_{\boldsymbol{s}'\in\mathcal{S}} P(\boldsymbol{s}'|\boldsymbol{s},\pi) \sum_{m=0}^{\infty} m\Pr[t_\pi(\boldsymbol{s}') = m-1] && (23)\\
&= \sum_{\boldsymbol{s}'\in\mathcal{S}} P(\boldsymbol{s}'|\boldsymbol{s},\pi) \sum_{m=-1}^{\infty} (m+1)\Pr[t_\pi(\boldsymbol{s}') = m] && (24)\\
&= \sum_{\boldsymbol{s}'\in\mathcal{S}} P(\boldsymbol{s}'|\boldsymbol{s},\pi) \sum_{m=0}^{\infty} (m+1)\Pr[t_\pi(\boldsymbol{s}') = m] && (25)\\
&= \sum_{\boldsymbol{s}'\in\mathcal{S}} P(\boldsymbol{s}'|\boldsymbol{s},\pi) \sum_{m=0}^{\infty} m\Pr[t_\pi(\boldsymbol{s}') = m] + \sum_{\boldsymbol{s}'\in\mathcal{S}} P(\boldsymbol{s}'|\boldsymbol{s},\pi) \sum_{m=0}^{\infty} \Pr[t_\pi(\boldsymbol{s}') = m] && (26)\\
&\overset{c}{=} \sum_{\boldsymbol{s}'\in\mathcal{S}} P(\boldsymbol{s}'|\boldsymbol{s},\pi) \sum_{m=0}^{\infty} m\Pr[t_\pi(\boldsymbol{s}') = m] + 1 && (27)\\
&\overset{d}{=} \sum_{\boldsymbol{s}'\in\mathcal{S}} P(\boldsymbol{s}'|\boldsymbol{s},\pi) T_\pi(\boldsymbol{s}') + 1, && (28)
\end{aligned}$$

with the following steps:

a. We apply Equation (19).

b. We apply Equation (20).

c. We employ the normalization of the probability distributions: $\sum_{m=0}^{\infty}\Pr[t_\pi(\boldsymbol{s}') = m] = 1$ and $\sum_{\boldsymbol{s}'\in\mathcal{S}} P(\boldsymbol{s}'|\boldsymbol{s},\pi) = 1$.

d. We use Equation (19) again.

In the previous derivation, we have implicitly assumed that the policy is deterministic: at each state $\boldsymbol{s}$, the action chosen is always $\pi(\boldsymbol{s})$. It can be shown that, in an MDP with a finite and countable set of states, there exists at least one optimal

policy that is deterministic (see Section 2.3 from [5]). Therefore, since we are solving a finite MDP, we only need to consider deterministic policies. Optimal random policies can be built by combining several deterministic optimal policies, provided that there is more than one.

When considering stochastic policies, π is no longer a mapping from a state to an action but a mapping from a state to a probability distribution over the action space. The previous derivation remains valid for stochastic policies, although in that case the transition probabilities must be written as

$$P(\boldsymbol{s}'|\boldsymbol{s},\pi) = \sum_{a\in\mathcal{A}} \pi(a|\boldsymbol{s}) P(\boldsymbol{s}'|\boldsymbol{s},a), \tag{29}$$

where $\mathcal{A}$ is the action space and $\pi(a|\boldsymbol{s})$ is the probability of choosing action a in state $\boldsymbol{s}$ when following policy π.

Supplementary Note 7. DYNAMIC PROGRAMMING ALGORITHMS

To find optimal policies, we formulate a Markov decision process that results in the Bellman equations, as explained in the main text. These equations can be solved using a dynamic programming algorithm, such as value iteration and policy iteration. Both algorithms start with arbitrary values of $T_\pi(\boldsymbol{s})$ (for some policy π and $\forall \boldsymbol{s} \in \mathcal{S}$, where $\mathcal{S}$ is the state space) and they iteratively update the policy π and the values $T_\pi(\boldsymbol{s})$, $\forall \boldsymbol{s} \in \mathcal{S}$. The updated policy is guaranteed to converge to an optimal policy π^* in a finite number of iterations in policy iteration and an infinite number of iterations in value iteration (see Sections 4.3 and 4.4 from [6]). Note that there might be multiple optimal policies, although this approach finds only one of them. In practice, the algorithms stop when the updated values differ by not more than some $\varepsilon > 0$ from the values in the previous iteration. All our results have been calculated using $\varepsilon = 10^{-7}$. In this work, we have applied both value iteration and policy iteration, which provided the same results (our specific implementations can be found at https://github.com/AlvaroGI/optimal-homogeneous-chain). For a detailed explanation of both algorithms, see Sections 4.3 and 4.4 from [6].

In terms of computational cost, policy iteration is generally faster since less iterations are required. To the best of our knowledge, there are no known tight bounds on the number of iterations until convergence. However, the computational complexity of a single iteration in policy iteration is $\mathcal{O}(|\mathcal{A}||\mathcal{S}|^2 + |\mathcal{S}|^3)$, where $\mathcal{A}$ is the action space and $\mathcal{S}$ is the state space, which can be prohibitive for some combinations of parameters [7]. The computational complexity of one iteration in value iteration is $\mathcal{O}(|\mathcal{A}||\mathcal{S}|^2)$ [7]. In our problem, the complexity of each iteration increases exponentially with increasing number of nodes and polynomially with increasing cutoff time (see Supplementary Note 9), and the number of iterations increases with decreasing probability of entanglement generation and decreasing probability of successful swap, since the estimate of the values is worse when these probabilities are small. Consequently, to study long chains with large cutoffs and small probabilities of successful entanglement generation and swap, one may need to employ approximate methods, such as deep reinforcement learning, which can find sub-optimal but good enough policies at a lower computational cost.

Supplementary Note 8. MARKOV DECISION PROCESS EXAMPLE

Here we provide an example of how to formulate the Markov decision process (MDP) for a three-node repeater chain with cutoff $t_{\mathrm{cut}} = 1$. Specifically, we calculate each term in the Bellman equations, which can then be used to find an optimal policy, as explained in the main text.

We start by listing all the states in which the chain can be found. The sequence of events during each time slot is the following:

1. First, the ages of all entangled links are increased by 1.
2. Second, entanglement generation is attempted between every pair of neighbors with qubits available.
3. Third, entanglement swaps can be performed.
4. Lastly, links whose age is equal to t_{cut} are removed. End-to-end links are not removed.

Since the cutoff is 1, the ages of all links are at most 1. All possible states are listed in Supplementary Figure 7.

Let us now find the equation for $T_\pi(\boldsymbol{s}_0)$, the expected delivery time from state $\boldsymbol{s}_0$, which is given by

$$T_\pi(\boldsymbol{s}_0) = 1 + \sum_{\boldsymbol{s}'\in\mathcal{S}} P(\boldsymbol{s}'|\boldsymbol{s}_0,\pi)\cdot T_\pi(\boldsymbol{s}'),$$

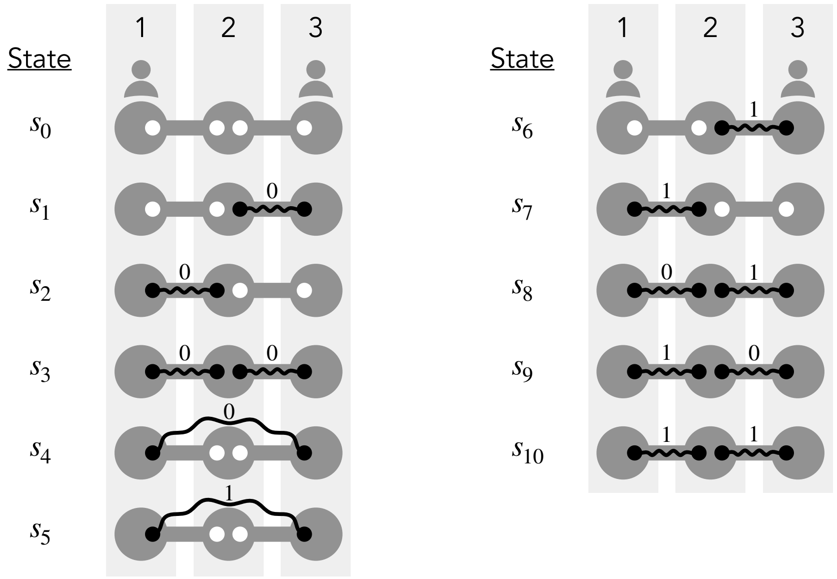

Supplementary Figure 7. All possible states in a three-node repeater chain with cutoff $t_{\mathrm{cut}} = 1$. Nodes are labeled 1 to 3 from left to right.

as explained in the main text and derived in Supplementary Note 6. For clarity, let us abuse notation and write T_i to denote $T_\pi(\boldsymbol{s}_i)$. We can find each term by considering each possible scenario separately:

- With probability $(1-p)^2$, no links are successfully generated and the state remains $\boldsymbol{s}_0$. Swaps and cutoffs do not apply to this state. This contributes with a term $(1-p)^2 T_0$.
- With probability $p(1-p)$, only one of the links is generated and the state becomes either $\boldsymbol{s}_1$ or $\boldsymbol{s}_2$. Swaps and cutoffs do not apply to these states. This contributes with $p(1-p)T_1 + p(1-p)T_2$.
- With probability p^2, both links are generated and the state becomes $\boldsymbol{s}_3$. Then, a swap can be performed and the last term splits into two contributions:
  - If the policy decides to perform a swap in node 2, i.e., $\pi(\boldsymbol{s}_3) = \{2\}$, the state at the end of the time slot will be $\boldsymbol{s}_4$ if the swap is successful, and $\boldsymbol{s}_0$ if the swap fails. These scenarios contribute with $p^2 \mathbb{1}_{\pi(\boldsymbol{s}_3)=\{2\}} \big(p_{\mathrm{s}} T_4 + (1-p_{\mathrm{s}}) T_0\big)$, where $\mathbb{1}_A$ is the indicator function that takes value 1 if A is true and value 0 otherwise.
  - If the policy decides to not perform the swap, i.e. $\pi(\boldsymbol{s}_3) = \emptyset$, the state remains $\boldsymbol{s}_3$. The contribution is then $p^2 \mathbb{1}_{\pi(\boldsymbol{s}_3)=\emptyset} T_3$.

Now, we can write all the terms above in a single equation:

$$T_0 = 1 + (1-p)^2 T_0 + p(1-p)T_1 + p(1-p)T_2 + p^2 \mathbb{1}_{\pi(\boldsymbol{s}_3)=\{2\}} \big(p_{\mathrm{s}} T_4 + (1-p_{\mathrm{s}}) T_0\big) + p^2 \mathbb{1}_{\pi(\boldsymbol{s}_3)=\emptyset} T_3. \tag{30}$$

Note that $T_4 = T_5 = 0$, since $\boldsymbol{s}_4$ and $\boldsymbol{s}_5$ are absorbing states. Rearranging terms, we obtain

$$T_0 = 1 + \Big[(1-p)^2 + p^2 \mathbb{1}_{\pi(\boldsymbol{s}_3)=\{2\}} (1-p_{\mathrm{s}})\Big] T_0 + \Big[p(1-p)\Big] T_1 + \Big[p(1-p)\Big] T_2 + \Big[p^2 \mathbb{1}_{\pi(\boldsymbol{s}_3)=\emptyset}\Big] T_3. \tag{31}$$

Let us now find the equation for $\boldsymbol{s}_1$. At the beginning of the time slot, the age of the link is increased by 1 and the state becomes $\boldsymbol{s}_6$. After that:

- With probability $(1-p)$, no links are successfully generated. Then, the only existing link is removed since it is 1 time slot old. This contributes with a term $(1-p)T_0$.
- With probability p, the remaining link is generated and the state becomes $\boldsymbol{s}_8$. Then, a swap can be performed:
  - If the policy decides to perform a swap in node 2, i.e., $\pi(\boldsymbol{s}_8) = \{2\}$, the state at the end of the time slot will be $\boldsymbol{s}_5$ if the swap is successful, and $\boldsymbol{s}_0$ if the swap fails. These scenarios contribute with $p \mathbb{1}_{\pi(\boldsymbol{s}_8)=\{2\}} \big(p_{\mathrm{s}} T_5 + (1-p_{\mathrm{s}}) T_0\big)$.
  - If the policy decides to not perform the swap, i.e. $\pi(\boldsymbol{s}_8) = \emptyset$, the state remains $\boldsymbol{s}_8$, and the link with age 1 is removed afterwards. The state becomes $\boldsymbol{s}_2$ and the contribution is then $p \mathbb{1}_{\pi(\boldsymbol{s}_8)=\emptyset} T_2$.

Combining all terms, we obtain

$$T_1 = 1 + \Big[(1-p) + p\mathbb{1}_{\pi(\boldsymbol{s}_8)=\{2\}}(1-p_\mathrm{s})\Big]T_0 + \Big[p\mathbb{1}_{\pi(\boldsymbol{s}_8)=\emptyset}\Big]T_2, \tag{32}$$

where we have used that $T_5 = 0$.

Due to the symmetry of the problem,

$$T_2 = T_1, \tag{33}$$

so we do not need to derive a new equation for T_2.

Lastly, we find the equation for $\boldsymbol{s}_3$. At the beginning of the time slot, the age of each link is increased by 1 and the state becomes $\boldsymbol{s}_{10}$, in which no more links can be generated. After that, a swap can be performed:

- If the policy decides to perform a swap in node 2, i.e., $\pi(\boldsymbol{s}_{10}) = \{2\}$, the state at the end of the time slot will be $\boldsymbol{s}_5$ if the swap is successful, and $\boldsymbol{s}_0$ if the swap fails. These scenarios contribute with $\mathbb{1}_{\pi(\boldsymbol{s}_{10})=\{2\}}\big(p_\mathrm{s}T_5 + (1-p_\mathrm{s})T_0\big)$.
- If the policy decides to not perform the swap, i.e. $\pi(\boldsymbol{s}_{10}) = \emptyset$, the state remains $\boldsymbol{s}_{10}$, and both links are removed afterwards, when cutoffs are applied. The state becomes $\boldsymbol{s}_0$ and the contribution is then $\mathbb{1}_{\pi(\boldsymbol{s}_{10})=\emptyset}T_0$.

The equation then reads

$$T_3 = 1 + \Big[\mathbb{1}_{\pi(\boldsymbol{s}_{10})=\{2\}}(1-p_\mathrm{s}) + \mathbb{1}_{\pi(\boldsymbol{s}_{10})=\emptyset}\Big]T_0, \tag{34}$$

where we have used that $T_5 = 0$.

We can write Equations (31), (32), (33), and (34) as

$$\begin{cases} T_0 = 1 + \Big[(1-p)^2 + p^2\mathbb{1}_{\pi(\boldsymbol{s}_3)=\{2\}}(1-p_\mathrm{s})\Big]T_0 + 2\Big[p(1-p)\Big]T_1 + \Big[p^2\mathbb{1}_{\pi(\boldsymbol{s}_3)=\emptyset}\Big]T_3, \\ T_1 = 1 + \Big[(1-p) + p\mathbb{1}_{\pi(\boldsymbol{s}_8)=\{2\}}(1-p_\mathrm{s})\Big]T_0 + \Big[p\mathbb{1}_{\pi(\boldsymbol{s}_8)=\emptyset}\Big]T_1, \\ T_3 = 1 + \Big[\mathbb{1}_{\pi(\boldsymbol{s}_{10})=\{2\}}(1-p_\mathrm{s}) + \mathbb{1}_{\pi(\boldsymbol{s}_{10})=\emptyset}\Big]T_0. \end{cases} \tag{35}$$

An optimal policy π^* can be found by minimizing T_0, T_1, and T_3 in this system of equations. This can be done, e.g., using iterative algorithms such as value and policy iteration, as discussed in the main text. In this case, it can be shown that the swap-asap policy is optimal, i.e., $\pi^*(\boldsymbol{s}_3) = \pi^*(\boldsymbol{s}_8) = \pi^*(\boldsymbol{s}_{10}) = \{2\}$. This also makes sense intuitively: once both links are generated, waiting provides no advantage in terms of delivery time over performing the swap immediately. For this policy, the system of equations becomes

$$\begin{cases} T_0 = 1 + \Big[(1-p)^2 + p^2(1-p_\mathrm{s})\Big]T_0 + 2\Big[p(1-p)\Big]T_1, \\ T_1 = 1 + \Big[(1-pp_\mathrm{s})\Big]T_0, \\ T_3 = 1 + \Big[(1-p_\mathrm{s})\Big]T_0, \end{cases} \tag{36}$$

which yields an optimal expected delivery time of

$$T_0 = \frac{1 + 2p(1-p)}{1 - (1-p)^2 - p^2(1-p_\mathrm{s}) - 2p(1-p)(1-pp_\mathrm{s})}.$$

As a final remark, note that the expected delivery times from states $\boldsymbol{s}_6$ to $\boldsymbol{s}_{10}$ were not necessary to compute T_0. In fact, states $\boldsymbol{s}_6$ to $\boldsymbol{s}_{10}$ cannot exist at the beginning of a time slot, since the links that exist at the beginning of a time slot are always younger than t_cut (i.e., their age is 0) or are end-to-end links. This is the reason why we do not need to optimize over T_6 to T_{10}.

Supplementary Note 9. SCALING OF THE NUMBER OF STATES

In this Supplementary Note, we find a lower bound to the number of states in the Markov decision process discussed in the main text, and show that it scales as $\Omega\big((t_\mathrm{cut})^{n-2}\big)$. Then, we compare this lower bound to the exact number of states for some combinations of parameters.

We start by calculating the lower bound. Let us define $\mathcal{S}(l)$ as the set of states in which only l entangled links are present. By definition,

$$\mathcal{S} = \bigcup_l \mathcal{S}(l).$$

The sets $\mathcal{S}(l)$ do not overlap. Therefore,

$$|\mathcal{S}| = \sum_l |\mathcal{S}(l)|. \tag{37}$$

The first term is given by

$$|\mathcal{S}(0)| = 1, \tag{38}$$

since there is only one state without any entangled links.

Since any two nodes could potentially share an entangled link, there are

$$k = \binom{n}{2} - 1 = \frac{n^2 - n - 2}{2}$$

possible links in a repeater chain with n nodes (note that we subtract 1 from the combinatorial number because there is no need to represent end-to-end links). When one of those links exists, its age can be anything from 0 to t_{cut}. Therefore, the total number of different states with only one link is given by

$$|\mathcal{S}(1)| = \frac{n^2 - n - 2}{2}(t_{\text{cut}} + 1) \geq \frac{n^2 - n - 2}{2} t_{\text{cut}}. \tag{39}$$

Let us now consider a chain with two links where both are connected to the same node i. From node i towards one end of the chain, there are $i - 1$ nodes. From i towards the other end of the chain, there are $n - i$ nodes. Then, the number of states with two links where both are connected to node i is given by

$$|\mathcal{S}_i(2)| = (i-1)(n-i)(t_{\text{cut}} + 1)^2,$$

where the last factor accounts for all possible ages of both links. We can find a lower bound to $|\mathcal{S}(2)|$ by considering only the states in which both links are connected to the same node i, i.e.,

$$\begin{aligned}
|\mathcal{S}(2)| &\geq \sum_{i=2}^{n-1} |\mathcal{S}_i(2)| \\
&= \sum_{i=2}^{n-1} (i-1)(n-i)(t_{\text{cut}} + 1)^2 \\
&= \sum_{j=1}^{n-2} j(n-j-1)(t_{\text{cut}} + 1)^2 \\
&= (t_{\text{cut}} + 1)^2 \sum_{j=1}^{n-2} \big(-j^2 + (n-1)j \big) \\
&= (t_{\text{cut}} + 1)^2 \frac{n(n-1)(n-2)}{6} \\
&\geq \frac{n(n-1)(n-2)}{6} t_{\text{cut}}^2,
\end{aligned} \tag{40}$$

where we used the identities $\sum_{k=1}^{m} k = \frac{m(m+1)}{2}$ and $\sum_{k=1}^{m} k^2 = \frac{m(m+1)(2m+1)}{6}$ to simplify the sum.

Next, we compute a lower bound for $|\mathcal{S}(l)|$, $l > 2$. Let us consider only states with l adjacent links, i.e., states that have $l - 1$ nodes that hold 2 entangled links. The number of such states is lower bounded by the different ways in which we can pick those $l - 1$ nodes from a total of $n - 2$ nodes (end-nodes cannot have two links) and the possible ages of the l links. Therefore, we can bound $|\mathcal{S}(l)|$ as follows:

$$|\mathcal{S}(l)| \geq \binom{n-2}{l-1} (t_{\text{cut}} + 1)^l \geq \binom{n-2}{l-1} t_{\text{cut}}^l, \quad l > 2. \tag{41}$$

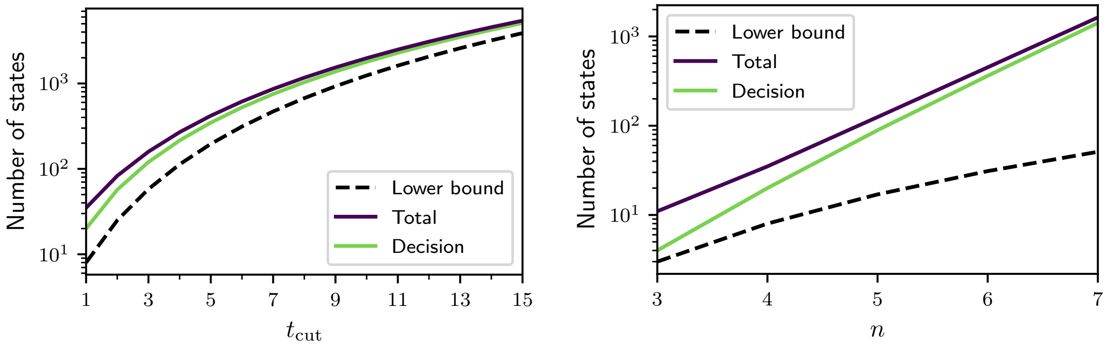

(a) **(b)**

Supplementary Figure 8. The number of states scales at least exponentially with increasing n and polynomially with increasing t_{cut}. (a) Number of states versus the cutoff time in a four-node chain, and (b) versus the number of nodes in a chain with cutoff $t_{\mathrm{cut}} = 1$. Solid lines correspond to the number of states found by our policy iteration algorithm (note that the number of states only depends on n and t_{cut}). The purple solid line is the total number of states and the green line is the number of states in which a decision can be made (i.e., states in which at least one swap can be performed). The dashed line corresponds to the lower bound (42) to the total number of states.

Finally, using Equations (37) to (41), we find a lower bound for $|\mathcal{S}|$:

$$\begin{aligned}
|\mathcal{S}| &= \sum_{l=0}^{2} |\mathcal{S}(l)| + \sum_{l=3}^{n-1} |\mathcal{S}(l)| \\
&\geq \sum_{l=0}^{2} |\mathcal{S}(l)| + \sum_{l=3}^{n-1} \binom{n-2}{l-1} t_{\mathrm{cut}}^{l} \\
&\geq \sum_{l=0}^{2} |\mathcal{S}(l)| + t_{\mathrm{cut}} \sum_{k=0}^{n-2} \binom{n-2}{k} t_{\mathrm{cut}}^{k} - t_{\mathrm{cut}} - (n-2)t_{\mathrm{cut}}^{2} \\
&\overset{a}{\geq} \sum_{l=0}^{2} |\mathcal{S}(l)| + t_{\mathrm{cut}}(t_{\mathrm{cut}}+1)^{n-2} - t_{\mathrm{cut}} - (n-2)t_{\mathrm{cut}}^{2} \\
&\geq \sum_{l=0}^{2} |\mathcal{S}(l)| + t_{\mathrm{cut}}^{n-1} - t_{\mathrm{cut}} - (n-2)t_{\mathrm{cut}}^{2} \\
&\geq 1 + \frac{n^2-n-2}{2} t_{\mathrm{cut}} + \frac{n(n-1)(n-2)}{6} t_{\mathrm{cut}}^{2} + t_{\mathrm{cut}}^{n-1} - t_{\mathrm{cut}} - (n-2)t_{\mathrm{cut}}^{2}
\end{aligned}$$

where, in step a, we have used the binomial sum:

$$\sum_{k=0}^{n} \binom{n}{k} x^k = (1+x)^n.$$

After some algebra, we find

$$|\mathcal{S}| \geq 1 + \frac{n^2-n-4}{2} t_{\mathrm{cut}} + \frac{(n^2-n-6)(n-2)}{6} t_{\mathrm{cut}}^{2} + t_{\mathrm{cut}}^{n-1}. \tag{42}$$

From the previous result, we conclude that the scaling of the number of states is

$$|\mathcal{S}| = \Omega\big((t_{\mathrm{cut}})^{n-1}\big).$$

Supplementary Figure 8 shows the exact number of states versus the cutoff time and the number of nodes, together with the lower bound (42). In these plots, the exact number of states corresponds to the size of the state space explored by our policy iteration algorithm.

Supplementary Note 10. CALCULATION OF TRANSITION PROBABILITIES WITH STATE BUNCHING

In this Supplementary Note, we explain how to simplify the calculation of transition probabilities with a technique that we call *state bunching*. This method takes advantage of state symmetries to reduce the number of equations and variables in the Bellman equations. Let us discuss how it works.

Definition 1. The mirrored version of a state is obtained by relabeling the nodes in reverse order. We denote the mirrored version of $\boldsymbol{s}$ as $\mathrm{mirror}(\boldsymbol{s})$.

Definition 2. A symmetric (sym) state $\boldsymbol{s}$ is one that is identical to its mirrored version, i.e., $\mathrm{mirror}(\boldsymbol{s}) = \boldsymbol{s}$. All other states are non-symmetric (non-sym).

Lemma 1. *Every non-sym state* $\boldsymbol{s}$ *has a mirrored version* $\tilde{\boldsymbol{s}}$ *that is different from* $\boldsymbol{s}$*, i.e.,*

$$\forall \boldsymbol{s} \in \mathcal{S} \;\; s.t. \;\; \boldsymbol{s} \neq mirror(\boldsymbol{s}), \;\; \exists\, \tilde{\boldsymbol{s}} \in \mathcal{S} \;\; s.t. \;\; mirror(\boldsymbol{s}) = \tilde{\boldsymbol{s}} \;\; and \;\; \boldsymbol{s} \neq \tilde{\boldsymbol{s}}.$$

The lemma above stems from the definition of non-sym state. Supplementary Figure 9 presents an example of non-sym state and its mirrored version.

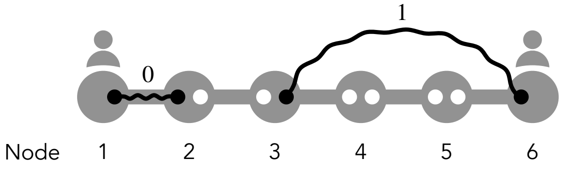

(a) An example state.

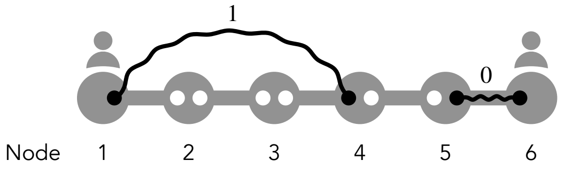

(b) The mirrored version of the state above.

Supplementary Figure 9. Example of a non-symmetric state and its mirrored version in a six-node repeater chain. Solid black lines represent entangled links, with occupied qubits in black and free qubits in white. The number above each entangled link corresponds to its age.

Let $\mathcal{S}$ be the set of states, $\mathcal{S}_{\mathrm{s}}$ be the set of sym states, and $\mathcal{S}_{\mathrm{ns}}$ be the set of non-sym states. Due to symmetry, the expected delivery times of a non-sym state and its mirrored version are equal, i.e., $T_\pi(\boldsymbol{s}) = T_\pi(\mathrm{mirror}(\boldsymbol{s}))$, for any policy π and any $\boldsymbol{s} \in \mathcal{S}_{\mathrm{ns}}$. Hence, we can remove half of the non-sym states from the Bellman equations to reduce the computational complexity, by following these steps:

1. Group all non-sym states in two sets, $\mathcal{S}_1$ and $\mathcal{S}_2$, such that neither set contains a state and its mirrored version, i.e., define $\mathcal{S}_1$ and $\mathcal{S}_2$ such that

$$\mathcal{S}_1 \cup \mathcal{S}_2 = \mathcal{S}_{\mathrm{ns}} \;\text{ and }\; \boldsymbol{s}_1 \neq \mathrm{mirror}(\boldsymbol{s}'_1), \forall \boldsymbol{s}_1, \boldsymbol{s}'_1 \in \mathcal{S}_1 \;\text{ and }\; \boldsymbol{s}_2 \neq \mathrm{mirror}(\boldsymbol{s}'_2), \forall \boldsymbol{s}_2, \boldsymbol{s}'_2 \in \mathcal{S}_2.$$

2. Replace $T_\pi(\boldsymbol{s}_2)$ by $T_\pi(\mathrm{mirror}(\boldsymbol{s}_2))$, for every $\boldsymbol{s}_2 \in \mathcal{S}_2$ in the Bellman equations. This can be done since $T_\pi(\boldsymbol{s}_2) = T_\pi(\mathrm{mirror}(\boldsymbol{s}_2))$. Note that $\mathrm{mirror}(\boldsymbol{s}_2) \in \mathcal{S}_1$.

3. The Bellman equations are now given by

$$T_\pi(\boldsymbol{s}) = 1 + \sum_{\boldsymbol{s}' \in \mathcal{S}_{\mathrm{s}} \cup \mathcal{S}_1} P(\boldsymbol{s}'|\boldsymbol{s}, \pi) \cdot T_\pi(\boldsymbol{s}') + \sum_{\boldsymbol{s}' \in \mathcal{S}_2} P(\boldsymbol{s}'|\boldsymbol{s}, \pi) \cdot T_\pi(\mathrm{mirror}(\boldsymbol{s}')), \; \forall \boldsymbol{s} \in \mathcal{S}_{\mathrm{s}} \cup \mathcal{S}_1,$$

which can also be written as

$$T_\pi(\boldsymbol{s}) = 1 + \sum_{\boldsymbol{s}' \in \mathcal{S}_{\mathrm{s}} \cup \mathcal{S}_1} \tilde{P}(\boldsymbol{s}'|\boldsymbol{s}, \pi) \cdot T_\pi(\boldsymbol{s}'), \; \forall \boldsymbol{s} \in \mathcal{S}_{\mathrm{s}} \cup \mathcal{S}_1, \tag{43}$$

where

$$\tilde{P}(\boldsymbol{s}'|\boldsymbol{s}, \pi) = \begin{cases} P(\boldsymbol{s}'|\boldsymbol{s}, \pi), & \text{if } \; \boldsymbol{s}' \in \mathcal{S}_{\mathrm{s}} \\ P(\boldsymbol{s}'|\boldsymbol{s}, \pi) + P(\mathrm{mirror}(\boldsymbol{s}')|\boldsymbol{s}, \pi), & \text{if } \; \boldsymbol{s}' \in \mathcal{S}_1 \end{cases}.$$

When using state bunching, we need to optimize a system of $|\mathcal{S}_\mathrm{s}| + |\mathcal{S}_1| < |\mathcal{S}|$ variables, as can be seen from (43).

Let us now assume that swaps are deterministic. With this assumption, we provide an analytical framework to simplify the calculation of the transition probabilities. Since there are several events happening in a single time slot, it is convenient to split P into two contributions. Each time slot can be divided in two parts:

A. The age of every link is updated (i.e., we add one to every link age), and then entanglement generation is attempted wherever qubits are available.

B. Swaps are performed according to action a, and then cutoffs are applied.

Definition 3. An intermediate state $\boldsymbol{r}$ is a state that exists between parts A and B of a time slot.

Supplementary Figure 10 presents an example of this division of the time slot for a six-node chain with cutoff $t_\mathrm{cut} = 2$. Let us define $P_\mathrm{A}(\boldsymbol{r}|\boldsymbol{s})$ as the probability that an intermediate state $\boldsymbol{r}$ is produced from an initial state $\boldsymbol{s}$ after the first part of the time slot. Note that P_A does not depend on any action, since swaps are performed after part A. Similarly, let us define $P_\mathrm{B}(\boldsymbol{s}'|\boldsymbol{r}, a)$ as the probability that the state at the end of the time slot is $\boldsymbol{s}'$ given that $\boldsymbol{r}$ was the intermediate state and action a was performed. We can write the transition probability from $\boldsymbol{s}$ to $\boldsymbol{s}'$ as follows:

$$P(\boldsymbol{s}'|\boldsymbol{s}, a) = \sum_{\boldsymbol{r}\in\mathcal{S}} P_\mathrm{B}(\boldsymbol{s}'|\boldsymbol{r}, a) P_\mathrm{A}(\boldsymbol{r}|\boldsymbol{s}).$$

Note that we only need to consider $\boldsymbol{s} \in \mathcal{S}_\mathrm{s} \cup \mathcal{S}_1$ to solve (43), while $\boldsymbol{s}', \boldsymbol{r} \in \mathcal{S}_\mathrm{s} \cup \mathcal{S}_1 \cup \mathcal{S}_2$. Next, we show that the calculation of P can be further simplified by ignoring some terms in the sum while adding some multipliers in other terms. We need the following definitions:

Definition 4. The *label of an entangled link* that is shared between nodes i and j is a tuple (i, j). Note that the label (i, j) is equivalent to (j, i).

Definition 5. Symmetry-preserving (SP) links of a repeater chain are those entangled links that retain their original labels in the mirrored version of a state. We denote all other links as non-symmetry-preserving (NSP).

Definition 6. The mirrored link of an NSP link with label (i, j) and age g is a link with label $(n - j + 1, n - i + 1)$ and age g.

If the transition during part A of the time slot is from a sym state $\boldsymbol{s}$ to a non-sym state $\boldsymbol{r} \in \mathcal{S}_1$, then a multiplier of two is necessary to omit states in $\mathcal{S}_2$. The reason is as follows: to transition to a non-sym state via entanglement generation, the chain must have generated an NSP link, and at the same time it must not have generated its mirrored link (the mirrored link can always be generated, since we start from a sym state). Note that generating an SP link does not affect the initial state's symmetry. Thus, the intermediate state $\boldsymbol{r}$ contains a new NSP link; however, had the chain generated its mirrored link instead, it would have generated mirror$(\boldsymbol{r}) \in \mathcal{S}_2$. Note that the probability of generating the NSP link is equal to that of generating its mirrored link, i.e., the transition probabilities to $\boldsymbol{r}$ and mirror$(\boldsymbol{r})$ are the same. Hence, the transition from $\boldsymbol{s}$ to mirror$(\boldsymbol{r}) \in \mathcal{S}_2$ can be captured by introducing a factor of two in $P_\mathrm{A}(\boldsymbol{r}|\boldsymbol{s})$. This way, we do not need to consider intermediate states $\boldsymbol{r}$ in $\mathcal{S}_2$ when $\boldsymbol{s}$ is a sym state.

Suppose now that the transition is from a sym state $\boldsymbol{s}$ to another sym state $\boldsymbol{r}$. Since both states are sym, the transition probability $P_\mathrm{A}(\boldsymbol{r}|\boldsymbol{s})$ is not affected when removing some non-sym states in state bunching.

Next, we note that transitions from a non-sym state $\boldsymbol{s}$ to a sym state $\boldsymbol{r}$ are not possible: if the initial state is non-sym, then it contains at least one NSP link without also containing its mirrored link. Such links would thus also be present in the intermediate state $\boldsymbol{r}$, with incremented ages, so that even if their mirrored links are generated, the new state would still be non-sym due to the age mismatch. It follows, then, that a non-sym state $\boldsymbol{s} \in \mathcal{S}_1$ may only transition to another non-sym state $\boldsymbol{r} \in \mathcal{S}_1$, and due to the presence of at least one NSP link with absence of its mirrored link, such a transition may only generate $\boldsymbol{r} \in \mathcal{S}_1$ and not mirror$(\boldsymbol{r}) \in \mathcal{S}_2$ (here, we assumed that $\boldsymbol{r}$ was placed in $\mathcal{S}_1$ when assigning non-sym states to each set; if this was not the case, we only need to exchange $\boldsymbol{r}$ and mirror$(\boldsymbol{r})$ to the opposite set). Thus, if both $\boldsymbol{s}$ and $\boldsymbol{r}$ are non-sym states, $P_\mathrm{A}(\boldsymbol{r}|\boldsymbol{s})$ is unaffected by state bunching. Supplementary Figure 10 depicts an example of such a transition.

Next, we shift our focus to $P_\mathrm{B}(\boldsymbol{s}'|\boldsymbol{r}, a)$, i.e., transitions that occur as a result of (deterministic) entanglement swaps, followed by link expirations. During part B of the time slot, state $\boldsymbol{r} \in \mathcal{S}_\mathrm{s} \cup \mathcal{S}_1$ may be taken to some state $\boldsymbol{s}'$ or mirror$(\boldsymbol{s}')$, depending on the combination of swaps. Take, for instance, the transition in Supplementary Figure 10: if the swap had been performed at the fourth node instead of the third, then the chain would have generated the mirrored version of the final state. Nevertheless, the chain must ultimately choose only one swapping combination at this stage. Since swaps and cutoffs are deterministic, either $\boldsymbol{s}'$ or mirror$(\boldsymbol{s}')$ (or none of them) is obtained deterministically. Therefore, there is no need to update $P_\mathrm{B}(\boldsymbol{s}'|\boldsymbol{r}, a)$ when using state bunching.

Finally, the transition probabilities can be written as

$$
\begin{aligned}
P(\boldsymbol{s}'|\boldsymbol{s},a) &= \sum_{\boldsymbol{r}\in\mathcal{S}_\mathrm{s}} P_\mathrm{B}(\boldsymbol{s}'|\boldsymbol{r},a)P_\mathrm{A}(\boldsymbol{r}|\boldsymbol{s}) + \sum_{\boldsymbol{r}\in\mathcal{S}_1} P_\mathrm{B}(\boldsymbol{s}'|\boldsymbol{r},a)\cdot 2\cdot P_\mathrm{A}(\boldsymbol{r}|\boldsymbol{s}), \text{ if } \boldsymbol{s}\in\mathcal{S}_\mathrm{s}, \\
P(\boldsymbol{s}'|\boldsymbol{s},a) &= \sum_{\boldsymbol{r}\in\mathcal{S}_1} P_\mathrm{B}(\boldsymbol{s}'|\boldsymbol{r},a)P_\mathrm{A}(\boldsymbol{r}|\boldsymbol{s}), \text{ if } \boldsymbol{s}\in\mathcal{S}_1,
\end{aligned} \tag{44}
$$

where we have removed all intermediate states from $\mathcal{S}_2$.

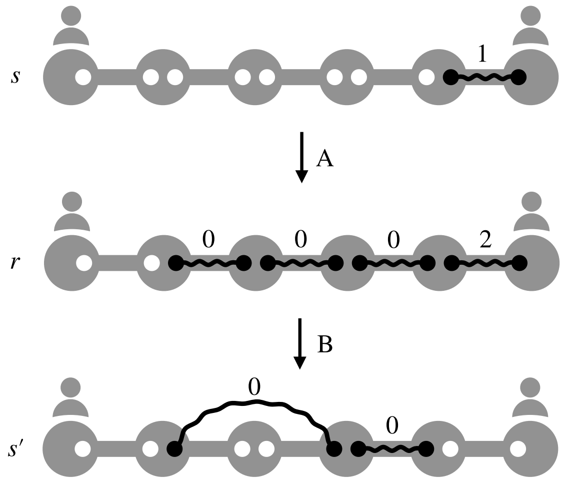

Supplementary Figure 10. Example of state evolution within a single time slot for a chain with six nodes and cutoff $t_\mathrm{cut} = 2$. The initial state is $\boldsymbol{s}$. During part A of the time slot, we update the age of every link and attempt entanglement generation, leading to an intermediate state $\boldsymbol{r}$. During part B of the time slot, swaps are performed and cutoffs are applied, leading to a final state $\boldsymbol{s}'$.

[1] Nielsen, M. A. & Chuang, I. *Quantum Computation and Quantum Information.* (Cambridge University Press, Cambridge, 2002).
[2] Dür, W., Hein, M., Cirac, J. I. & Briegel, H. J. Standard forms of noisy quantum operations via depolarization. *Phys. Rev. A*, **72**, 052326 (2005).
[3] Munro, W. J., Azuma, K., Tamaki, K. & Nemoto, K. Inside quantum repeaters. *IEEE Journal of Selected Topics in Quantum Electronics*, **21**, 78–90 (2015).
[4] Briegel, H. J., Dür, W., Cirac, J. I. & Zoller, P. Quantum repeaters for communication. Preprint at https://arxiv.org/abs/quant-ph/9803056 (1998).
[5] Szepesvári, C. Algorithms for reinforcement learning. *Synthesis lectures on Artificial Intelligence and Machine Learning*, **4**, 1–103 (2010).
[6] Sutton, R. S. & Barto, A. G. *Reinforcement Learning: an Introduction.* (MIT press, Cambridge, 2018).
[7] Kaelbling, L. P., Littman, M. L. & Moore, A. W. Reinforcement learning: a survey. *J. of Art. Intell. Res.*, **4**, 237–285 (1996).
